# MinION Analysis and Reference Consortium: Phase 1 data release and analysis

**DOI:** 10.12688/f1000research.7201.1

**Published:** 2015-10-15

**Authors:** Camilla L.C. Ip, Matthew Loose, John R. Tyson, Mariateresa de Cesare, Bonnie L. Brown, Miten Jain, Richard M. Leggett, David A. Eccles, Vadim Zalunin, John M. Urban, Paolo Piazza, Rory J. Bowden, Benedict Paten, Solomon Mwaigwisya, Elizabeth M. Batty, Jared T. Simpson, Terrance P. Snutch, Ewan Birney, David Buck, Sara Goodwin, Hans J. Jansen, Justin O'Grady, Hugh E. Olsen, MinION Analysis and Reference Consortium

**Affiliations:** 1 Wellcome Trust Centre for Human Genetics, University of Oxford, Oxford, UK; 2 School of Life Sciences, Queens Medical Centre, University of Nottingham, Nottingham, UK; 3 Michael Smith Laboratories and Djavad Mowafaghian Centre for Brain Health, University of British Columbia, Vancouver, Canada; 4 Virginia Commonwealth University, Richmond, VA, USA; 5 University of California, Santa Cruz, Santa Cruz, CA, USA; 6 The Genome Analysis Centre, Norwich Research Park, Norwich, UK; 7 Malaghan Institute of Medical Research, Wellington, New Zealand; 8 European Molecular Biology Laboratory, European Bioinformatics Institute, Wellcome Trust Genome Campus, Hinxton, UK; 9 Division of Biology and Medicine, Brown University, Providence, RI, USA; 10 Norwich Medical School, University of East Anglia, Norwich, UK; 11 Informatics and Biocomputing, Ontario Institute for Cancer Research, ON, Canada; 12 Cold Spring Harbor Laboratory, Cold Spring Harbor, NY, USA; 13 ZF-screens B.V., Leiden, Netherlands

**Keywords:** MinION, nanopore sequencing, data release, long reads, minoTour, marginAlign, NanoOK, third-generation sequencing

## Abstract

The advent of a miniaturized DNA sequencing device with a high-throughput contextual sequencing capability embodies the next generation of large scale sequencing tools. The MinION™ Access Programme (MAP) was initiated by Oxford Nanopore Technologies™ in April 2014, giving public access to their USB-attached miniature sequencing device. The MinION Analysis and Reference Consortium (MARC) was formed by a subset of MAP participants, with the aim of evaluating and providing standard protocols and reference data to the community. Envisaged as a multi-phased project, this study provides the global community with the Phase 1 data from MARC, where the reproducibility of the performance of the MinION was evaluated at multiple sites. Five laboratories on two continents generated data using a control strain of *Escherichia coli* K-12, preparing and sequencing samples according to a revised ONT protocol. Here, we provide the details of the protocol used, along with a preliminary analysis of the characteristics of typical runs including the consistency, rate, volume and quality of data produced. Further analysis of the Phase 1 data presented here, and additional experiments in Phase 2 of *E. coli* from MARC are already underway to identify ways to improve and enhance MinION performance.

## Introduction

The idea of using nanopores as biosensors was suggested by several groups starting in the 1990s (patent by Church *et al.*, submitted 1995, published 1998; Kasianowicz *et al.*, 1996). Investigators documented that ionic current passing through a nanopore depends on the identity of nucleic acid bases interacting with and transiting the nanopore ( Akeson *et al*., 1999; Derrington *et al.*, 2010; Manrao *et al.*, 2011; Wallace *et al*., 2010). Nanopores were also found able to resolve the order of bases in nucleic acid molecules ( Akeson *et al.*, 1999; Bayley *et al.*, 2006; Laszlo *et al.,* 2014; Song *et al*., 1996). A final key step leading to sequencing was reduction of DNA translocation speed through the nanopore using enzymatic control (e.g., polymerase) to feed the nucleic acid strand to the pore, base-by-base, on a millisecond time scale ( Cherf *et al.*, 2012; Lieberman, 2010). Oxford Nanopore Technologies ( https://www.nanoporetech.com) was founded in 2005 to translate these proof-of-concept studies into a commercial third-generation sequencing device. The announcement of the MinION, a device that can detect bases of a single-stranded DNA (ssDNA) molecule that passes through a nanopore with no theoretical limits on read length (except those introduced during sample preparation), was met with enthusiasm at the Advances in Genome Biology and Technology (AGBT) meeting in 2012 ( Check Hayden, 2012; Eisenstein, 2012). Independent beta-testing of the MinION device began in April 2014 with the start of the MinION Access Programme (MAP) ( https://www.nanoporetech.com/community/the-minion-access-programme) involving over 1,000 laboratories. The first publications appeared in late 2014 and early 2015 ( Ammar *et al.*, 2015; Ashton *et al.*, 2015; Greninger *et al.*, 2015; Jain *et al.*, 2015; Karlsson *et al.*, 2015; Kilianski *et al.*, 2015; Laver *et al.*, 2015; Loman *et al.*, 2015; Mulley & Hargreaves, 2015; Quick *et al.*, 2014; Urban *et al.*, 2015; Wang *et al.*, 2015) and these provided a first glimpse of the performance characteristics and limitations of the device at that time, as well as potential applications.

**Figure 1.**
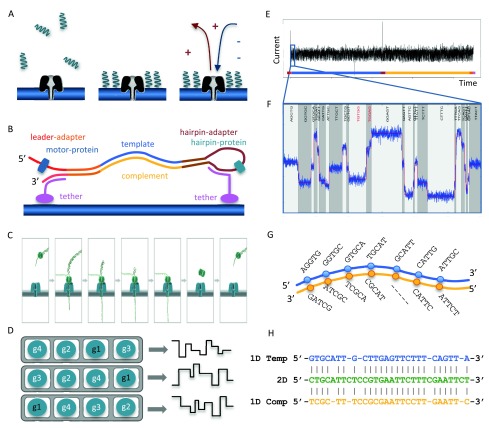
The Oxford Nanopore sequencing process. ( **A**) Suspended library molecules are concentrated near nanopores embedded in the membrane. A voltage applied across the membrane induces a current through the nanopores. ( **B**) Schematic of a library molecule, showing dsDNA ligated to a leader adapter pre-loaded with a motor protein and a hairpin adapter pre-loaded with a hairpin protein, and the tethering oligos. ( **C**) Sequencing starts from the 5’ end of the leader adapter. The motor protein unwinds the dsDNA allowing single-stranded DNA to pass through the pore. ( **D**) A flow cell contains 512 channels (grey), each channel consisting of 4 wells (white). Each well contains a pore (blue) and a sensor. At any given time, the device is recording the data stream from the wells of the active well-group, in this example, g1. ( **E**) Perturbation in the current across the nanopore is measured 3,000 times per second as ssDNA passes through the nanopore. ( **F**) The ‘bulk data’ are segmented into discrete ‘events’ of similar consecutive measurements. The 5-mer corresponding to each event is inferred using a statistical model. ( **G**) The 1D base-calls are inferred separately for the template and complement event signals. ( **H**) Alignment of the 2D base-calls from the event signals from both, and the 1D base-calls are used to constrain the 2D base-calls.

The MinION is the smallest high-throughput sequencing platform available to date: a 90g device, 10 cm in length, that is able to sequence individual molecules of DNA with a single-use flow cell. To enable sequencing of both strands, a library is constructed from double-stranded DNA (dsDNA) with a protocol similar to that used for short-read, second-generation platforms. The library preparation chemistries (SQK–MAP005 and SQK–MAP005.1) used in this study, contain two different adapters that are ligated to the DNA ( Figure 1A). The first, the ‘leader adapter’, consists of two oligos with partial complementarity that form a Y-shaped structure once annealed. The second, the ‘hairpin adapter’, is a single oligo with internal complementarity to form a hairpin structure. Both adapters in the sequencing kit used for this study are preloaded with ‘motor proteins’ that mediate the movement of DNA through the pore. Another function of the adapters is to guide the DNA fragments to the vicinity of pores via binding to tethering oligos with affinity for the polymer membrane ( Figure 1B). Sequencing begins at the single-stranded 5’ end of the leader adapter ( Figure 1C). Once the complementary (double-stranded) region of the leader adapter is reached, the motor protein loaded onto the leader adapter unzips the dsDNA, allowing the first strand of the DNA fragment, the ‘template’, to be passed into the nanopore one base at a time, while the sensor measures changes in the ionic current. After reaching the hairpin adapter, an additional protein, the ‘hairpin protein’, allows the complementary strand of DNA to pass through the nanopore in a similar fashion. The current MinION flow cell has 512 channels, each connected to 4 wells which may each contain a nanometer-scale biological pore (nanopore) embedded in an electrically-resistant membrane bilayer ( Figure 1D). Each channel provides data from one of the four wells at a time, the order of use defined by the allocation of wells to well-groups during an initial ‘mux scan’ ( File S2 Glossary), allowing up to 512 independent DNA molecules to be sequenced simultaneously.

When a voltage is applied across the membrane, an ion current flows through the nanopore. The translocation of ssDNA through the nanopore causes a drop in the current that is characteristic of the bases in contact with the pore at that time ( Figure 1E, Laszlo *et al.*, 2014). A sensor measures the current in the nanopore several thousand times per second (at 3,000Hz in this study), the data streams are passed to the ASIC (application-specific integrated circuit) and the MinKNOW software. The raw current measurements are compressed into a sequence of ‘events’, each being a mean current value with an associated variance and duration ( Figure 1F). The raw current measurements or the corresponding events, plotted over time, are referred to as a ‘squiggle plot’. The base-caller in use at this time modelled the characteristics of 4 ^5^ (= 1,024) possible 5-mers and base-calling consisted of finding the optimal path ( Figure 1G) through a Hidden Markov Model (HMM) of successive 5-mers using a Viterbi algorithm ( http://www.bio-itworld.com/news/02/17/12/Oxford-strikes-first-in-DNA-sequencing-nanopore-wars.html). The 1D base-calls are inferred separately for the template and complement event signals ( Figure 1G), the 2D base-calls from the event signals from both, and the 1D base-calls are used to constrain the 2D base-calls ( Figure 1H).

The release of version R7+ flow cells by Oxford Nanopore to the MAP community provided highly positive feedback concerning both utility and quality of the MinION data. However, it became clear that groups were having different degrees of success with the MinION, with the possible influencing factors being difficult to infer from a single sequencing run. The MARC Phase 1 experiments were designed to assess the yield, accuracy, and reproducibility of MinION data by undertaking replicate experiments across multiple sites, with the intention of identifying technical factors important for consistently high performance. To this end, five laboratories initially sequenced the same *Escherichia coli* strain K-12 substrain MG1655, in duplicate, using a single shared protocol for culture, extraction of high-quality total genomic DNA, library preparation and sequencing ( File S1). A laboratory *E. coli* strain was chosen as it has a single circular chromosome of 4.6 Mb that could be sequenced to sufficient depth in a single MinION run and a complete reference sequence is available ( NCBI RefSeq NC_000913). The detailed protocol for sequencing double-stranded total genomic DNA was based on the standard protocol from ONT at the time the experiment was conceived. During the generation of the sequencing data for this work, referred to here as the Phase 1a experiments, updates to the ONT sequencing kit and protocol were made available (version MN005_1124_revC_02Mar2015, last modified 10 June 2015, https://wiki.nanoporetech.com/pages/viewpage.action?pageId=28246488). To ensure this study included data from these updates, we generated an equivalent dataset using the updated protocol, referred to here as the Phase 1b experiments.

An initial lack of tools for the analysis of data obliged the MAP community to develop a series of bioinformatics solutions for exploring the native FAST5 data ( Table S2) produced by the MinION. Poretools ( Loman & Quinlan, 2014, https://github.com/arq5x/poretools) and poRe ( Watson *et al.*, 2015, http://sourceforge.net/projects/rpore/) are packages for converting and visualising the raw data, whereas minoTour ( http://minotour.github.io/minoTour) provides real-time analysis and control of a sequencing run and post-run analytics. NanoOK ( Leggett *et al.*, 2015, https://documentation.tgac.ac.uk/display/NANOOK/NanoOK) uses alignment-based methods to assess quality, yield, and accuracy of the data. New software packages such as marginAlign ( Jain *et al*., 2015, https://github.com/benedictpaten/marginAlign), NanoCorr ( Goodwin *et al.*, 2015, http://schatzlab.cshl.edu/data/nanocorr/), Nanopolish ( Loman *et al.,* 2015, https://github.com/jts/nanopolish/) and PoreSeq ( Szalay & Golovchenko, 2015, https://github.com/tszalay/poreseq) were developed to address the relatively high error rate of the raw data and allow genome assembly and error-correction from MinION reads. Some of these tools were used for the MARC Phase 1 data analyses.

At the time of this writing, around a dozen reports have emerged recounting utility of the MinION for *de novo* sequencing of viral, bacterial, and eukaryotic genomes. The MinION data from this study constitute the only resource, to date, of carefully replicated experiments across multiple laboratories that can be used to infer the volume, quality and reproducibility of data from the platform. At the time the Phase 1 experiments were run, extensive preliminary analysis revealed clear factors influencing site-to-site reproducibility and provided inspiration for future MARC experiments in which we will explore improvements to the MinION sequencing protocol.

## Materials and methods

Each group used the following protocols to obtain total genomic DNA from freshly grown cells, fragment the DNA, prepare libraries, and sequence the libraries using the MinION. The full methods are described in the supplementary information ( File S1).

### Culture of *the E. coli* K-12 target sample

To remove variability that might be caused by freeze-thaw of genomic DNA and based on previous observations that fresh material gave better results, each group worked with freshly prepared total genomic DNA from *E. coli* str. K-12 substr. MG1655 purchased from DSMZ, Germany ( https://www.dsmz.de, DSM No. 18039) on 21 January 2015. On arrival, the *E. coli* strain was rehydrated in LB broth. The rehydrated culture was used to inoculate ten replicate 10 mL LB broth tubes and one plate, all of which were incubated overnight at 37°C. Following incubation, the plate was examined to ensure the culture was pure. Broth cultures were centrifuged at 5,000 × g in a benchtop centrifuge to collect biomass for cryogenic bead tube (Protect, Lab M, Lancashire, UK) inoculation. Bead tubes were stored at -70°C until they were shipped, at room temperature, to four other laboratories ( Table S1). Upon arrival, the bacterial culture was plated on LB agar, checked for viability and purity, and the bead tube stored at -80°C until the sample was ready for culture and extraction.

### DNA extraction and library preparation

At each participating laboratory, DNA was extracted from approximately 4 × 10 ^9^ log-phase cells using QIAGEN Genomic-tip 20/G according to the manufacturer’s instructions (QIAGEN, Valencia, California). A library was prepared the day after extraction using the Genomic DNA Sequencing Kit SQK–MAP005 according to the base protocol from Oxford Nanopore (version MN005_1123_revA_02Mar2015) with slight modifications from the MARC consortium ( File S1).

In summary, genomic DNA (1 µg and 1.5 µg for the Phase 1a and 1b experiments, respectively) was fragmented using Covaris g-TUBE (Covaris, Ltd., Brighton, United Kingdom) to achieve a fragment distribution with a peak at ~10 Kb (3,300 × g). The sheared DNA was pretreated with PreCR Repair Mix (New England Biolabs, Ipswich, Massachusetts) to repair possible damage to the DNA that could interfere with the sequencing process: since the DNA passes through the pore as a single strand, the presence of a nick is of particular concern because it would prematurely terminate the sequencing of the molecule. To protect the DNA from further damage during the preparation of the library, vortexing was avoided and more gentle mixing approaches (i.e., pipetting, inverting, or gentle flicking) were used instead. After clean-up with 1× AMPure XP beads (Beckman Coulter, Brea, California) to remove PreCR reagents from the sample, the DNA was resuspended in fresh 10 mM Tris-HCl pH 8.5, and concentration and fragment size were assessed using the Qubit dsDNA BR assay (Life Technologies, Grand Island, New York) and the Agilent TapeStation where available (Agilent Technologies, Santa Clara, California). In Phase 1a, all remaining genomic DNA was used for the next stage while in Phase 1b, which started with 1.5 µg, 1 µg of the genomic DNA remaining at this point was used. For most libraries, an internal control DNA sequence (‘DNA CS’ from the SQK–MAP005 kit, corresponding to the last ~3,555 bases of Enterobacteria phage lambda, RefSeq NC_001416.1, with a single mutation G45352A) was added at this point. The DNA was then prepared using the NEBNext End Repair Module, cleaned with 1× AMPure beads, treated with the NEBNext dA-Tailing Module (New England Biolabs) and cleaned again with 1× AMPure beads prior to ligation.

The final ligation of adapter and hairpin was performed in Protein LoBind 1.5 ml tubes (to avoid loss of protein-loaded adapters) with Blunt/TA Ligase Master Mix (New England Biolabs) followed by a pulldown step using his-tag Dynabeads (Life Technologies). Extra care was taken to mix reagents during the ligation and following steps only through careful pipetting, so to avoid unnecessary contact of the ligated and protein-bound DNA with the tube walls.

### Sequencer configuration and sequencing run conditions

The MinION device is controlled by the MinKNOW™ Software Agent on the connected computer. The Metrichor™ Desktop Agent manages the connection to the base-calling service in the cloud hosted by Metrichor. Installation of the most current version of software for both programs at the time of each experiment was strictly enforced. Thus, the software versions used to process different experiments were highly correlated with the date on which experiments were commenced. While the library was being prepared, the MinION device was made ready for sequencing. A new R7.3 flow cell, provided to the MARC Phase 1 laboratories from the same lot number, was fastened to the MinION device and the MinKNOW Platform QC recipe script was run to assess the number of pores in each channel available for sequencing. A minimum of 400 g1 channels (of a possible 512) was considered acceptable. At the end of the QC, the flow cell was primed/washed twice ( File S1, steps 79–80) and the sequencing run started after loading the library (6 µl for Phase 1a runs or 12 µl for Phase 1b runs). Once the 48-hour sequencing recipe script had been initiated, the Metrichor Desktop Agent was started and the raw data files were automatically uploaded to the Metrichor cloud-based service for base-calling. To maximize the yield of higher quality sequence data from the device, an additional aliquot of stored library (that had been held at 4°C) was loaded at 24h to coincide with the pre-set g1-to-g2 pore switch to fresh wells with active pores.

### Base-calling and data formats

The output of the MAP_48Hr_Sequencing_Run script is one FAST5 file per read. The FAST5 format ( File S2) used by Oxford Nanopore is a variant of the HDF5 standard ( File S2, https://www.hdfgroup.org) with a hierarchical internal structure designed to store the metadata associated with the sequencing of that read and the events (aggregated bulk current measurements) pre-processed by the MinION ( Table S2). The data from each instance of the MAP_48Hr_Sequencing_Run script are allocated a ‘run number’ (referred to in this study as the ‘batch id’, File S2 Glossary), and within this batch, each read is produced by one of the 512 channels and numbered by a ‘file number’ starting from zero. The combination of experiment name, batch, channel and file number is sufficient to uniquely identify a read. During the Phase 1 experiments, a 128-bit numerical universally-unique identifier (UUID) ( https://tools.ietf.org/html/draft-mealling-uuid-urn-03), represented as a 32-digit hexadecimal number, was introduced to the FAST5 format as an alternate unique identifier for each read.

The FAST5 file for each read is uploaded to the cloud base-calling service by the Metrichor agent, base-calls are inferred, the read is allocated to a ‘read class’ of either ‘pass’ or ‘fail’ based on the criteria used at the time ( File S2). All the data in the raw FAST5 plus additional metadata and the base-calls themselves are packaged into a base-called FAST5 file ( Table S2) with a more complex internal structure and downloaded to the ‘pass’ or ‘fail’ subfolder of the pre-specified ‘downloads’ directory on the client computer. At the time the Phase 1 experiments were performed and base-called, the read class could only be inferred from the directory in which it was deposited by Metrichor.

### ENA data pre-processing pipeline

The base-called FAST5 files and associated metadata from each of the five labs and 20 experiments were collated on a server at the European Nucleotide Archive (ENA, http://www.ebi.ac.uk/ena) and run through a bespoke pipeline of pre-processing tools ( Table S3). The ENA pipeline extracted the 2D base-calls from the base-called FAST5 files with poreTools version 0.5.1 ( Loman & Quinlan, 2014), then aligned every read to the *E. coli K-12* reference genome (NCBI RefSeq Accession NC_000913.1) using BWA-MEM version 0.7.12-41044 with the nanopore data parameters ‘-× ont2d’ ( Li, 2013) and LAST version 460 ( Kielbasa *et al*., 2011). Both the BWA-MEM and the LAST alignments were post-processed using marginAlign version 0.1 ( Jain *et al*., 2015). Statistics on each of the four alignments were computed by SAMtools version 1.2 ( Li *et al*., 2009), poreMap version 0.1.1 ( https://github.com/camilla-ip/poremap), marginStats ( Jain *et al*., 2015), and identity version 0.1 ( https://github.com/enasequence/ONT). The number of target, control and unclassified reads produced during each experiment was inferred by mapping each 2D read to the *E. coli* and lambda reference sequences, then allocating each read to either target or control when there was a single significant alignment to the respective genome. The remaining reads were recorded as ‘unclassified’ if they mapped to both or neither of the possible references. A consensus sequence of the nanopore reads mapped to the appropriate *E. coli* reference was inferred by Nanopolish version 0.3.0 ( Loman *et al.,* 2015) and included with the analyses as part of the data release.

All base-called FAST5 files and the outputs of the ENA pipeline for the 20 experiments ( Table S10) are available through ENA project PRJEB11008 ( http://www.ebi.ac.uk/ena/data/view/PRJEB11008).

### Data analyses

In this study, we describe the data that match the chronological order in which they were generated and processed, from raw events, to 1D, then 2D base-calls. We then explored accuracy, at each stage, quantifying the data produced under the standard MARC protocol and commenting on how variations from that protocol may have affected the data yield or accuracy. Preliminary analyses of the data relied on summaries and visualisations from the minoTour webserver ( http://minotour.github.io/minoTour), reports generated by NanoOK version 0.54 ( Leggett *et al.*, 2015), and bespoke Python and R scripts. To explore variations over time, each read was allocated to the 15 minute interval in which the read commenced sequencing, the number of active pores (where an active pore was defined as one that was still producing reads), and the read counts were converted to number of reads per hour per active pore. Plots were generated by allocating the events from each read to the appropriate 15 min interval under the assumption that events are produced at a steady rate for each read. The percentage of the 512 active pores in each window was then computed, normalising event yield by the number of active pores to derive the event rate in events per hour per pore. The median read length in events was computed for the reads from each experiment commencing in each 15 min interval. Reads generated from the first 1h, between 24 and 25h, and the last 1h of the experiments were not shown as the flow cell characteristics determining the data generation rate were obscured by stochastic effects arising from the initiation, well switching, and low active pore numbers toward the end of each experiment. The default run script does not attempt to base-call reads with less than 200 or more than 230,000 events, the arbitrary limits originally introduced to limit the memory requirements of the base-caller. To reduce noise that would otherwise obscure the underlying degradation rate of the flow cell chemistry, reads outside the callable length range were excluded and although the ‘Basecaller XL’ workflow currently available can call reads with up to 1 million events, we did not attempt to base-call these extra long reads in this study. The final figures, tables, and supplementary material were based on summary statistics for every read from every experiment generated by poreQC version 0.2.10 ( https://github.com/camilla-ip/poreqc) and poreMap version 0.1.1 ( https://github.com/camilla-ip/poremap).

The spike-in of a control sample of known DNA is useful for calibrating the accuracy of data from an experiment, especially when a good reference sequence for the target sample is not available. Ideally, sufficient control sample reads would be obtained to perform these analyses, but not so many that the yield of target sample is significantly diminished. Thus, the proportion of reads that are from the target rather than the control sample is another metric that affects the usable yield of the MinION. The proportion of target and control reads in each sample was inferred by NanoOK, which mapped each 2D read to the *E. coli* and lambda reference sequences using BWA-MEM ‘-× ont2d’, and classified each read to the genome of the primary alignment, or reported the read as ‘unclassified’.

To quantify the error rate of reads produced by the MinION and explore the effect that different alignment methods, metrics and read types have on the values reported, we produced an error metric we refer to as ‘total percent error’ of a read; that is, the percentage of a read that is inaccurate due to miscalled bases, inserted bases in the read, and deleted bases that are missing from the read but present in the reference sequence. The intent of this approach was to circumvent alignment-dependent biases that may reduce the miscall rate at the expense of insertions and deletions (indels).

Since the accuracy metrics are computed from alignments of base-calls to the appropriate reference and each alignment method used will produce slightly different estimates, we computed the total error, and the components, for four alignment strategies: initial alignment by BWA-MEM (parameters ‘-× ont2d’) or LAST (parameters ‘-s 2 -T 0 -Q 0 -a 1’, as recommended by Quick *et al.*, 2014), followed by re-alignment with marginAlign ( Jain *et al.*, 2015), which uses expectation maximization to train an HMM and estimate Maximum Likelihood Estimation (MLE) parameters that are, in turn, used to infer higher confidence alignments guided by the AMAP objective function (Schwartz *et al*., 2007). The alignment-based calculations provided by minoTour, NanoOK and poreMap were based on BWA-MEM (parameters ‘-× ont2d’). Further data processing was performed by bespoke Python scripts and extracts of the data plotted using either bespoke R scripts or minoTour. For clarity, the data and algorithm used to derive each figure are described briefly at the appropriate point in the Results section.

Sequencing bias of the MinION was explored with the over- and under-represented 5-mer table produced by NanoOK. If a platform is capable of sequencing any DNA sequence, all possible 5-mers in the DNA should be proportionally represented in the data when counts are normalized for the distribution of all 5-mers in the genome. Thus, the most under-represented and over-represented 5-mers in the base-calls from the MinION may suggest limitations or biases of the nanopore sequencing process. The NanoOK tables were computed from a hash table of read k-mer counts generated by moving a sliding window of size 5 base-by-base over each FASTQ read and counting 5-mers. The relative abundance of each read k-mer was calculated by dividing the k-mer count by the total number of k-mers in all the reads. Similarly, a hash table of reference 5-mer counts was generated from the reference sequence. The most under-represented 5-mers were deemed to be those with the largest difference in relative abundance between the reads and reference and where the reference abundance was greater than the read abundance. The most over-represented 5-mers were deemed to be those with the largest difference in relative abundance between the reads and the reference and where the read abundance was greater than the reference abundance.

## Results

### Experimental design

A total of 20 experiments (individual flow cell runs) were performed in two stages (Phase 1a and 1b) by five laboratories. Experiments from Phase 1a and 1b used the SQK–MAP005 and SQK–MAP005.1 Genomic DNA Sequencing Kits, respectively, which required a template mass of 1 µg and 1.5 µg, and library volume of 6 µl and 12 µl, respectively. Each laboratory ( Table S1) undertook two identical replicate experiments for each kit version. The 20 experiments are henceforth referred to as P1a-Lab1-R1 to P1b-Lab5-R2, following a ‘phase-lab-replicate’ format.

### Variation in DNA concentration and template lengths

The Phase 1a and Phase 1b experiments started with an *E. coli* template DNA mass of 1 μg and 1.5 μg, respectively. A fraction was lost during each clean up step of the library preparation protocol so that after fragmentation, end repair, and dA-tailing, only 17% of the Phase 1a and 29% of the Phase 1b starting DNA was retained ( Table S4). Measurements of the P1a-Lab4-R1 DNA size distribution revealed a peak at ~15 Kb that subsequently translated into a typical read-length distribution, suggesting that the read length achieved by the MinION closely resembles the length of the input DNA fragments.

### Variation in library preparation

Most Phase 1a experiments deviated at least once from the standard protocol during the library preparation steps. Variations included starting with a higher DNA mass, the suspected addition of an incorrect concentration of fuel mix, skipping addition of the DNA CS (lambda phage control spike-in sample) DNA, and using a higher library volume ( Table S5). Phase 1b experiments experienced less unplanned variation.

### Variability in initial flow cell quality

**Figure 2.**
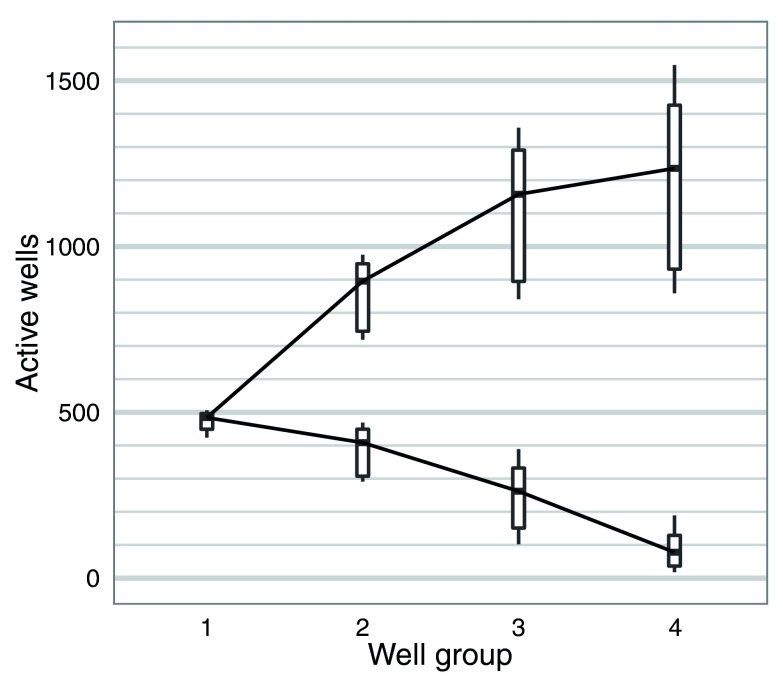
Initial active pore count. The distribution of the number of active pores (lower series) and the cumulative total (upper series) for well-groups 1 to 4 are shown for the 20 flow cells used in this study. The measurement for each experiment was made either during the Platform QC or at the beginning of the 48h script.

The number of active pores in each of the four well-groups was measured once during the Platform QC (-180 mV) (steps 52-53, File S1) before sequencing commenced and at the start of the 48h sequencing protocol (-140 mV) (step 87, File S1), and one of these measurements was recorded for each flow cell ( Table S4). Although the numbers reported by the Platform QC are higher than mux numbers from the 48H script, possibly due to the different bias voltage used by the two scripts, either value gives a good indication of initial flow cell quality. The median number of active pores reported across the experiments was 484, 409, 262 and 78 for well-groups g1 to g4, respectively, which corresponds to 95, 80, 51 and 15% of the theoretical maximum of 512 each ( Figure 2). The standard sequencing protocol only utilizes the first two well-groups during a run. Thus, although on average 60% of the 2,048 wells contained an active pore, only 44% of all pores in a typical flow cell were available for sequencing utilizing the standard sequencing protocol ( Figure 2).

### Uniformity of sequencing software used during the experiments

All Phase 1a and 1b experiments were performed over a period of about one month each, between 27 March and 27 April 2015, and between 15 and 21 April 2015 , respectively ( Table S6). Comparison of the sequencing-related attributes stored in the FAST5 files ( Table S2) confirmed that most parameters were identical among and between the Phase 1a and 1b experiments, the exceptions being minor variations in the versions of the MinKNOW and Metrichor Desktop Agents, the Oxford Nanopore sequencing protocol and the event detection software ( Table 1, Table S7).

**Table 1.** Differences in sequencing software across the Phase 1 experiments.

Software	Phase 1a version	Phase 1b version
MinKNOW	0.49.2.9	0.49.2.9, 0.49.3.7
Metrichor	0.10.0	1.12.1
Protocols	0.49.2.9, 0.49.2.11	0.49.2.11, 0.49.3.7
ONT sequencing workflow	0.10.1	0.10.2
Event detection	0.49.2.9	0.49.2.9, 0.49.3.7
chimaera (analysis software pipelines)	0.10.1	0.12.2

### Variation due to forced restarts of the sequencing protocol script

Once started, the MAP_48Hr_Sequencing_Run protocol performs a ‘Platform QC’ to allocate active pores to well-groups 1 through to 4, starts sequencing with the active pores in well-group 1, switches to use the active pores in well-group 2 at 24h, and automatically terminates at 48h. However, the sequencing protocol was aborted or restarted at least once for 5 of the Phase 1a and 3 of the Phase 1b experiments because: (i) the number of active pores and the data yield were so low that the user decided to discontinue the run without a restart (N=4); (ii) the sequencing computer crashed (N=1); or (iii) the hard drive filled up (N=3) ( Table 2, Table S8). While the sequencer was being restarted, there was usually a period when it was idle, explaining differences between the total sequencing time and the total time over which the device was active (c.f. seq_duration_hrs and run_duration_hrs, Table S8). In addition, 6 of the 20 sequencing experiments were restarted at 48h ( Table 2, Table S8) to test whether the device can continue to produce good data beyond the standard 48h script provided by Oxford Nanopore, but all such data were excluded from this analysis.

**Table 2.** Data listing. Adherence to the standard wet-lab protocol for each batch, and the start number and well-group origins of reads produced in each experiment.

	Phase 1a											Phase 1b											
	Lab1		Lab2		Lab3		Lab4		Lab5		All	Lab1		Lab2		Lab3		Lab4		Lab5		All	All
	R1	R2	R1	R2	R1	R2	R1	R2	R1	R2		R1	R2	R1	R2	R1	R2	R1	R2	R1	R2		
standard wet-lab	✔		✔	✔			✔				4		✔	✔	✔	✔	✔	✔	✔			7	11
Start1, g1	✔	✔	✔	✔	✔	✔	✔	✔	✔	✔	10	✔	✔	✔	✔	✔	✔	✔	✔	✔	✔	10	20
Start1, g2	✔		✔	✔	✔	✔	✔	✔	✔	✔	9		✔	✔	✔		✔	✔	✔	✔	✔	8	17
Start2, g1		✔			✔	✔				✔	4	✔	✔									3	7
Start2, g2										✔	1											0	1
Start3, g1		✔			✔						2	✔										1	3
Start3, g2											0	✔										1	1
Std data	✔		✔	✔							3		✔	✔	✔			✔	✔			5	8
Std data >48h			✔	✔							2			✔	✔			✔	✔			4	6

### Data exclusion

Despite the existence of a detailed standard protocol, a number of method deviations were recorded arising variously from wet-lab omissions or errors, flow cell quality issues, and computer software and hardware issues ( Table S5). Thus, we could not use all the data generated to infer the yield, accuracy, and variability produced by a MinION because of the variations among the 20 experiments ( Table 2, Table S5). Eleven of the experiments (P1a: N=4; P1b: N=7) adhered precisely to the wet-lab component of the standard MARC protocol; the other 9 contained at least one variation, mostly due to uncontrollable factors ( Table 2, Table S5). Therefore, data was restricted to reads generated during the first execution of the MAP_48Hr_Sequencing_Run script (held in common among experiments) and those generated under common, near-standard conditions. With this strategy, we avoided unusual data accumulation patterns resulting from experiment restarts, which results in well swapping via pore reselection (re-mux), while still taking advantage of all 20 experiments, even those that terminated before 48h due to computer failures or flow cell issues. Each start of the MAP_48Hr_Sequencing_Run protocol generates one batch of data, with up to ½ h being from flow cell calibration and mux pore selection, the next 24h being from the first well-group pores, and the remainder from the second well-group pores. We generated reads from the g1 and g2 well-group pores of the first start of 20 and 17 experiments, respectively. Of the 7 experiments that started the sequencing protocol for a second time, 7 generated data from the g1 well-group pores and 1 from the g2 well-group pores. Similarly, for the 3 experiments that had a third start, 3 experiments generated data from the g1 well-group pores and 1 from the g2 well-group pores ( Table 2).

### Temperature regulation of the flow cell

Anecdotal reports from MAP participants have suggested that the temperature of the flow cell can affect the performance and data quality of the MinION. In our experiments, each flow cell operated at a characteristic temperature with only minor fluctuations over time. All flow cells had an ASIC temperature between 23.9 and 35.2 **°**C (median 26.8 **°**C) and a heat-sink temperature of 36.8 to 38.6 **°**C (median 37.0 **°**C). There was no correlation between the DNA input mass or fuel amount and the resulting operating temperature, and temperatures observed during Phases 1a and 1b were similar. The flow cells with the highest yields, P1a-Lab3-R1 and P1b-Lab4-R1, had ASIC temperatures that spanned the range observed (26.9 **°**C and 35.2 **°**C, respectively), suggesting that operating temperature does not tend to affect data yield.

### Total event yield

**Figure 3.**
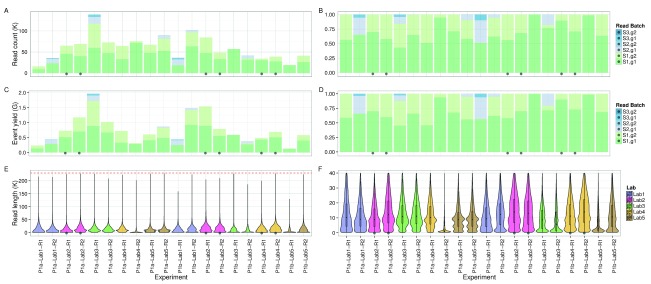
Event yield for 20 experiments. Read count as ( **A**) raw counts and ( **B**) a percentage. Event yield as ( **C**) raw counts and ( **D**) a percentage. The ( **E**) entire distribution of callable read lengths and ( **F**) a subset showing the lower part in more detail. The 6 experiments that adhered to the MARC protocol and sequenced for at least 46h are marked with a black dot. The upper callable threshold of 230,000 events is indicated by a red dashed line.

If the deviations from the established protocol can be considered as corresponding to normal variation in use, examination of the total data produced by the 20 Phase 1 experiments provides an indication of the total yield that can be expected from the current platform. We found a high level of variability among the 20 experiments that was only partially attributable to protocol deviations: a median of 60,600 reads (inter-quartile range (IQR) of 38,000 to 74,000, max. 139,000) ( Figure 3A,B) containing 650,000 events (IQR 434,000 to 750,000, max. 1.9 million) ( Figure 3C,D). Very few (~0.2%) of the events were in reads that were not base-called by Metrichor because they were outside the pre-set callable length range of 200 events to 230,000 events.

**Figure 4.**
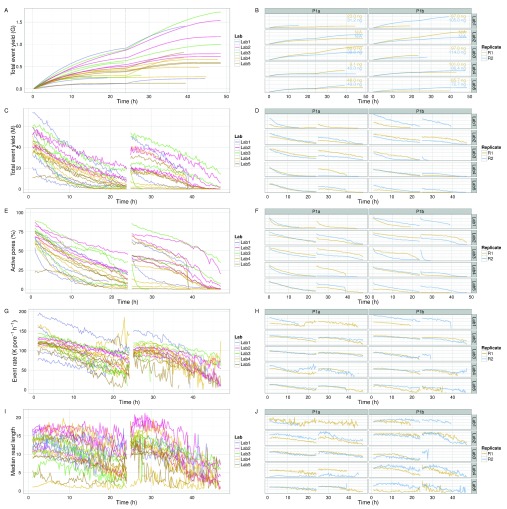
Event generation profile. ( **A**, **B**) Cumulative event yield. ( **C**, **D**) Event yield per hour. ( **E**, **F**) Percentage of the 512 pores that were active. ( **G**, **H**) Event sequencing rate per pore. ( **I**, **J**) Length of reads in events. The left plots show the values for each experiment, coloured by lab. The right plots show the values for each experiment more clearly. The DNA input mass for each experiment is provided in ( **B**). Data collected during the first hour, the hour following the pore-group switch (24–25h) and the last hour (47–48h) are omitted for clarity.

**Figure 5.**
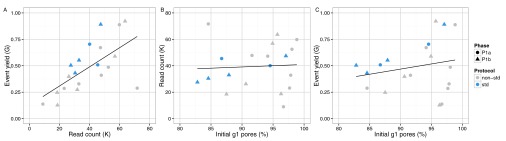
Relationship between number of initial g1 pores, read count and event yield. The phase of the experiment is indicated by shape. The experiments that adhered to the MARC protocol for both the wet-lab and sequencing components are shown in blue.

**Figure S1.**
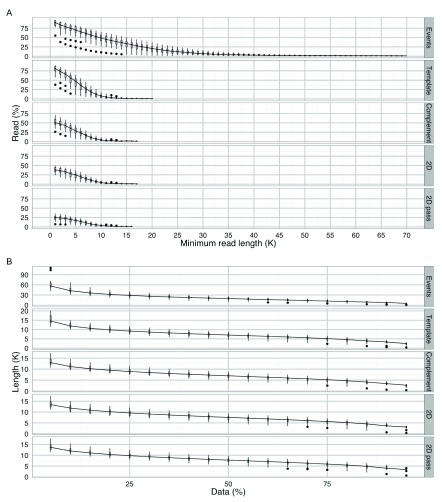
Proportion of long reads and data in long reads. ( **A**) The percentage of reads with a length greater than a specified read length. A boxplot of the percentage of reads was plotted for each read in multiples of 1000 until the read percentage dropped to 1%. Typically, 21% of the reads had a length of over 20,000 events, and 7.6%, 4.0%, 4.4% and 3.6% of the reads had over 10,000 bases in the template, complement, 2D and 2D ‘pass’ base-calls. ( **B**) The length of reads containing a specified percentage of the data. Typically, 50% of the reads had a length of at least 13,600 events, and 5,500, 5,600, 6,000 and 6,300 bases for the template, complement, 2D and 2D ‘pass’ base-calls. Similarly, 5% of the reads had a length of at least 56,600 events, and 14,500, 13,000, 13,500 and 13,600 bases for the template, complement, 2D and 2D ‘pass’ base-calls.

The median read lengths from the 20 experiments indicate most experiments had a broad distribution with a peak around 10,700 events and a long tail containing a very small number of reads that reached the upper limit of 230,000 events ( Figure 3E,F). Typically, a median of 20% of the reads had a length of at least 21,000 events ( Figure S1A), and 50% of the events were in reads of at least 13,600 events, 25% of the events were in reads of at least 29,000 events, and 5% in reads of at least 56,600 events ( Figure S1B). The event generation rate was not constant during a sequencing run. Of the 9 experiments that ran for at least 46h, 67% of the events were produced in the first 24h ( Figure 4A,B). Although a higher read count is associated with a higher event yield ( Figure 5A), neither the number of reads nor the event yield was strongly correlated with the number of active g1 pores ( Figure 5B,C), suggesting data yield is not solely dependent on the number of initial active pores. Although the experiments that followed the MARC wet-lab protocol precisely (blue triangles, Figure 5) had a higher event yield to read count and higher event yield to initial g1 pore count, the effect was not large and does not form a distinguishable cluster among the rest of the experiments.

To evaluate whether the variation could be due to deviations from the MARC protocol, we examined event data generated by the g1 pores of the first start of all 20 experiments, all of which ran for at least 23 hours. No significant relationship was found between the total read count, total event yield or event lengths and the input DNA mass (Pearson’s correlation coefficient, p=0.036, 0.221 and 0.149, respectively). Similarly, the Kruskal-Wallis test found no significant difference between the number of reads, total event yield, or median event lengths between the Phase 1a and 1b experiments (p=0.290, 0.151 and 0.482, respectively), the five labs (p=0.482, 0.159 and 0.263, respectively), or the 6 experiments that strictly adhered to the MARC protocol and the remainder of the experiments (p=0.909, 0.183, and 0.119, respectively).

The highest data yield was from experiment P1a-Lab3-R1, which commenced sequencing with the highest number of active g1 pores (506/512 = 98.8%) to produce over 138 thousand reads and almost 2 billion (1×10 ^9^) events within the callable read length range ( Table S4, Table S6). The library for this experiment contained a DNA input mass of 60 ng in 12 μL of PSM, which was less than the median of 70 ng across the 20 experiments ( Table S6 Experiments). That the two experiments with the highest event yield (P1a-Lab3-R1 and P1b-Lab4-R1) used a lower mass of input DNA (60 ng and 9.1 ng, respectively), confirms that the amount of DNA loaded is greater than that required to keep the active pores adequately supplied with DNA molecules.

Experiment P1a-Lab3-R2 was notable in that it was run for almost 62h, first for 48h using the standard sequencing script, then for an additional 8.1h and 4.8h with two starts of a modification of the MAP_48Hr_Sequencing_Run recipe script, MAP_2×8hrs_180_190_Sequencing_Run.py that performs a new allocation of wells to well-groups (re-mux) ( File S2) well selection followed by 8h of sequencing at each of -180 mV and -190 mV, respectively (SQK–MAP005 script developed by John Tyson available to the MAP community at https://wiki.nanoporetech.com/x/tgLDAQ). During the extra 15h, the total accumulated yield increased by 8% ( Table S6, Table S8), demonstrating that good flow cells can continue to produce significant amounts of data with the appropriate software.

### Event yield profile over time

**Figure S2.**
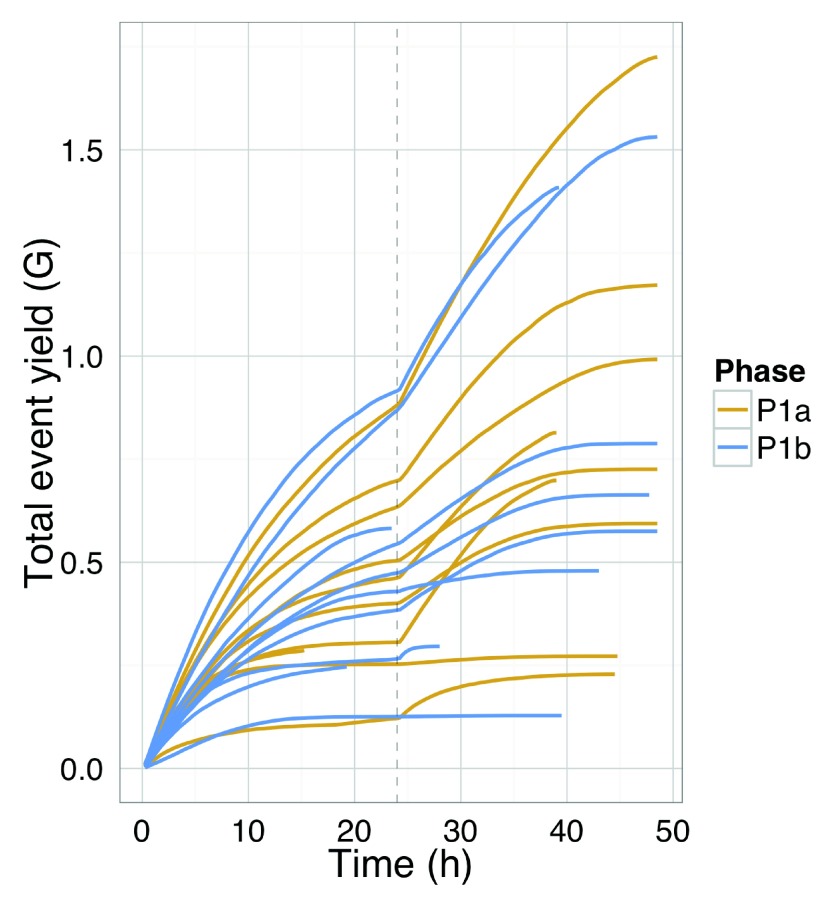
Cumulative event yield over time by phase. The cumulative event yield was plotted for all reads from each experiment and coloured by the experimental phase. The yield for each phase has not affected the rate of event production over time or the total events produced. The total yield was not dependent on the input DNA mass (for input DNA mass, see SI Table 4). The re-mux and library reload at 24h is shown by a vertical dashed line.

**Figure S3.**
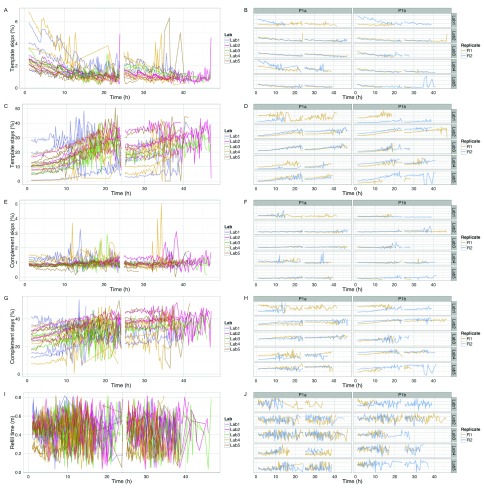
Additional factors affecting event yield over time. ( **A**, **B**) Percentage of template events that are skips (i.e., event moves with a step length > 1). ( **C**, **D**) Percentage of template events that are stays (i.e., event moves with a step length = 0). ( **E**, **F**) Percentage of complement events that are skips (i.e., event moves with a step length > 1). ( **G**, **H**) Percentage of complement events that are stays (i.e., event moves with a step length = 0). ( **I**, **J**) Mean number of minutes that a pore is idle between sequencing instances. The number of skip and stay events was inferred for the template and complement strands of each reads and allocated to the 15 minute interval since the start of the experiment. The refill plot was based on values computed with poreQC version 0.2.10. The number of seconds the pore was idle before sequencing commenced was computed for each read, excluding the first read and any read which followed a read which did not result in a valid set of events, allocated to the 15 minute window in which the read commenced, grouped by experiment, and the median plotted for each experiment. Only data for the first sequencing script start is shown. The first hour, the hour following the pore-group switch and the last hour, are not shown for clarity. The 24h re-mux and library reload is shown by a vertical dashed line.

All experiments demonstrated event accumulation rates that decreased for the first 24h, experienced a sharp increase at 24h following the pore group switch and library reload, then steadily decreased again until the run was terminated ( Figure 4A). There was no obvious correlation between total yield and input DNA ( Figure 4B), lab ( Figure 4B), or phase ( Figure S2). The flow cells commenced sequencing at 120–200 × 10 ^3^ events h ^-1^ ( Figure 4G,H). Although the experiments generated between 0.2 and 1.2 billion events ( Figure 4A), a typical run such as P1b-Lab2-R2 generated 47% of the data (367 million events) in the first quarter (12h) of the experiment and 69% of the data (544 million events) in the first half (24h) of the experiment ( Figure 4B). The rate at which events accumulated over time in each experiment was similar ( Figure 4), suggesting a shared mechanism. The decrease in event yield over time ( Figure 4C,D) correlates with a decrease in the number of active pores ( Figure 4E,F). However, the decreasing number of pores cannot be the sole determining factor as even when normalized for the number of active pores, the event yield still declined over time approximately linearly for the first 24h (with the exception of P1b-Lab4-R2), then less predictably for the next 24h ( Figure 4G,H). The decrease in event length over time may be another contributing factor ( Figure 4I,J), but the pore refill delay, or the time during which pores are idle, appears constant during a run ( Figure S3I,J). The sequence of 5-mers inferred from a sequence of events may suggest that a base of the library molecule being sequenced has been skipped (e.g., a skip of 1 base may be inferred from a progression from AATGC to TGCCG) or that a base has been sequenced more than once (e.g., a stay may be inferred from consecutive 5-mers AATGC and AATGC). While we hypothesized that a decrease in events over time may be caused by an increase in skips and stays, we observed a decrease in the percentage of template skips ( Figure S3A,B) but a lower and constant percentage of complement skips ( Figure S3E,F), and an increase in template and complement stays over time ( Figure S3C,D,G,H). In conjunction with 4h periodic effects in the plots (e.g., SI Figure 3B, P1a-Lab2-R1/R2), this suggests an increasing stay rate, possibly due to non-optimal bias voltage across the flow cell membrane, may be a contributing factor to the lower event rate observed during an experiment, and this phenomenon would benefit from further investigation. Another point to note is that the profiles of experiments produced at the same lab are more similar to each other than to experiments from other labs ( Figure 4 and Figure S3, right side plots), suggesting lab effects or the MinION device may be contributing to the effect.

### Proportion of target and control sample

**Figure 6.**
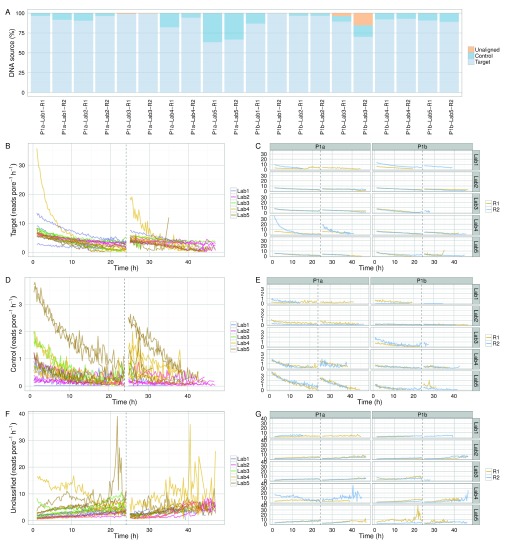
Read yield of target and control samples. ( **A**) Proportion of target, control and unclassified 2D reads for each experiment. The read production rate (reads pore ^-1^ h ^-1^) for ( **B**, **C**) target DNA, ( **E**, **F**) control DNA, and ( **F**, **G**) reads that could not be aligned uniquely either to the target or control reference sequence.

Between 63% and 99% (median 92%) of the reads were allocated to the target sample and most of the remainder to the control sample ( Figure 6A). Two Phase 1a experiments omitted to include the control sample (P1a-Lab3-R1 and P1a-Lab3-R2) ( Figure 6A). Phase 1b experiments P1b-Lab3-R1 and P1b-Lab3-R2 contained a larger proportion of reads (3.7% and 15.3%, respectively) that did not map to either the target or the control reference ( Figure 6A), suggesting contamination. Taxonomic classification of all 2D reads using Kraken version 0.10.5-beta ( Wood & Salzberg, 2014) found only two experiments with non- *E. coli* bacterial matches: P1b-Lab3-R1 had 2.3% of the reads classified as Pseudomonadales (probably *Pseudomonas putida*) and P1b-Lab3-R2 had 10.7% of reads as Pseudomonales (probably *P. putida*) and 2.2% as Burkholderiales (best match sp. *P. delftia*), species implicated in kit contamination ( Salter *et al.*, 2014) at percentages comparable to those inferred from the BWA-MEM alignments.

With the exception of outlier experiments from P1a-Lab4-R2 (that may have been run with extra initial fuel) and P1a-Lab5 (which, for reasons unknown, sequenced DNA at a higher rate than in other experiments), the proportion of target and control reads decreased at a similar rate, suggesting the platform was not biased towards either ( Figure 6B–E). The increasing rate of unclassifiable reads over time ( Figure 6F,G) likely reflects decreasing read quality over time.

### Yield and quality of 1D and 2D base-calls of the target sample

**Figure 7.**
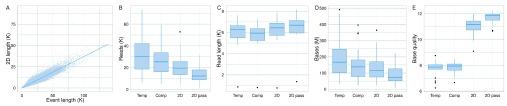
Summary of 1D and 2D base-calls. ( **A**) The relationship between event lengths and the length of 2D base-calls is linear, with a slope of 0.367 (ratio of 2.7 : 1). The distribution of ( **B**) total number of reads, ( **C**) read length; ( **D**) total base yield; and ( **E**) mean base quality of the target sample across the 20 experiments.

The length of events and 2D base-calls of all target reads from all experiments had a linear relationship with a slope of 0.367 (ratio of 2.7 : 1) ( Figure 7A). The median numbers of template, complement, 2D, and 2D ‘pass’ reads across the 20 experiments were 30,360, 25,370, 19,540 and 12,320 bases, respectively ( Figure 7B); the median read lengths were 6,280, 5,940, 6,440 and 6,690 bases, respectively ( Figure 7C); the median base yields were 167, 137, 115 and 74 million bases, respectively ( Figure 7D); the median base yield of each type was 167, 138, 115 and 73 million bases, respectively; and the median of mean base quality of the base-calls of each type was 7.9, 7.9, 11.2 and 11.9, respectively ( Figure 7E).

**Figure 8.**
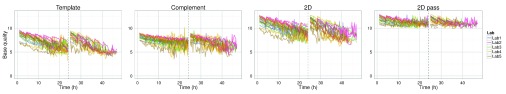
Base quality variation over time for 1D and 2D base-calls of the target sample. The median base quality for template, complement, all 2D, and 2D pass bases in 15 minute intervals for target DNA reads. Statistics are inferred from data from the first start of each sequencing experiment. Data collected during the first hour, the hour following the pore-group switch (24–25h) and the last hour (47–48h) are omitted for clarity.

**Figure 9.**
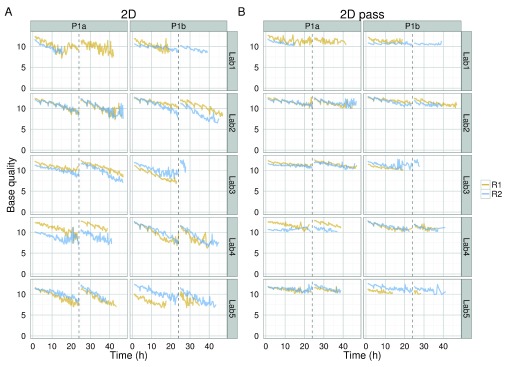
Variation in base quality of 2D and 2D pass base-calls during an experiment. The mean base quality for 15 minute intervals for ( **A**) all 2D reads and ( **B**) 2D pass reads in each experiment.

**Figure S4.**
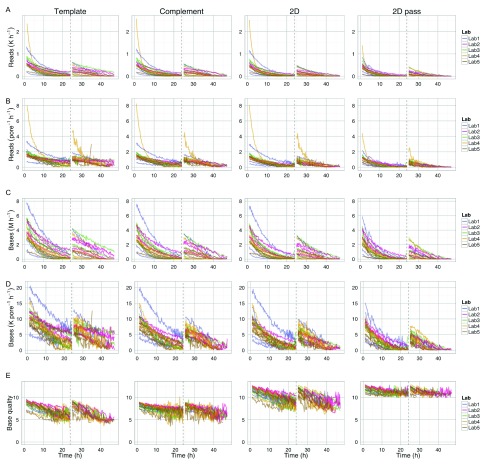
Yield and quality of 1D and 2D base-calls over time. Each row shows ( **A**) read count, ( **B**) read count per pore per hour, ( **C**) base yield, ( **D**) base yield per pore per hour, and ( **E**) base quality for 1D template and complement reads, 2D reads and the 2D ‘pass’ reads. The values were inferred from the statistics computed by poreQC version 0.2.10 and poreMap version 0.1.1. Only data for the first sequencing script start is shown. The first hour, the hour following the pore-group switch and the last hour, are not shown for clarity. The 24h re-mux and library reload time is shown by a vertical dashed line.

Not only did the rate of read production decrease over time for all 1D and 2D reads ( Figure S4A–D), all experiments also exhibited a declining trend in base quality over time ( Figure 8 and Figure 9, Figure S4E). The template, complement, and 2D bases differed from the start of each sequencing run, having a mean base quality of about 2 units less after 24h of sequencing ( Figure 8 and Figure 9). The increase in the rate of read production ( Figure 4) at the 24h mux switch was accompanied by an increase in the base quality ( Figure 8). Every 4h, there was a smaller-scale recapitulation of the decline followed by a return in base quality, most clearly seen in the P1b-Lab2 experiments ( Figure 8 and Figure 9, Figure S4), coinciding with the -5 mV bias-voltage adjustment every 4h in the 48h sequencing protocol script (mux1 voltage sequence (mV): -140, -145, -150, -155, -160, -165; followed by mux2 voltage sequence (mV): -155, -160, -165, -170, -175, -180) to maintain a more uniform current flow.

**Figure 10.**
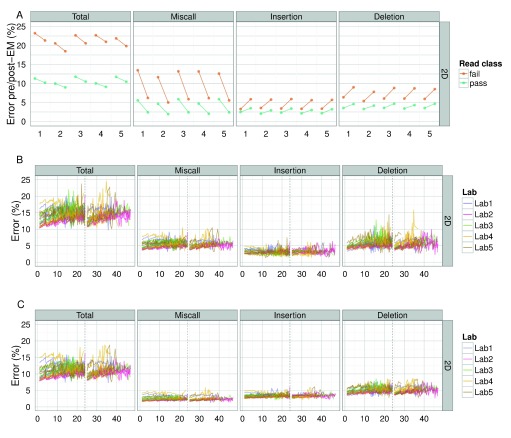
Effect of EM correction on BWA-MEM alignments of target 2D base-calls. ( **A**) The total percentage error of each read, grouped by laboratory, for values computed from BWA-MEM alignments pre- and post-EM correction; ( **B**) the median percentage error over time for alignments by BWA-MEM for each experiment; and ( **C**) the median percentage error over time for alignments by BWA-MEM followed by EM correction for each experiment, showing the median total, miscall, insertion and deletion error for each 15 minute interval.

**Figure S5.**
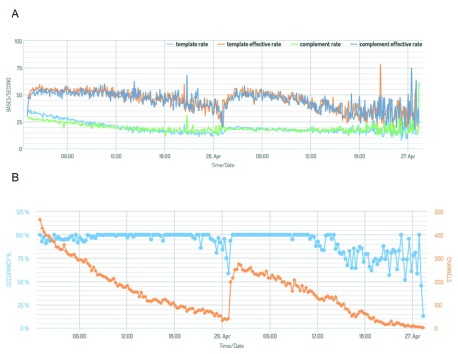
Sequencing rate and pore occupancy rate for a typical experiment. ( **A**) Mean read sequencing rate of the template (light blue) and complement bespoke (green) strands, measured in bases per second, for each 15 minute interval for experiment P1a-Lab2-R2. The effective sequencing rate, computed as the total time taken to sequence bases the template and complement bases, per unit time, per active channel are shown for the template (orange) and complement (dark blue) for the same 15 minute intervals. In a typical experiment like P1a-Lab2-R2, template and complement sequences were produced at a declining rate over the course of 24h, and for both metrics, the rate at which template sequences translocate through the pore decreases more rapidly than the complement sequences. ( **B**) The percentage of time that active pores were occupied (blue, left axis) and the number of active channels across the device (orange, right axis, maximum of 512), for 15 minute intervals during experiment P1a-Lab2-R2. Active pores continued to produce data at a similar rate until they became inactive, which happened at a relatively uniform rate during an experiment.

To investigate the interplay between sequencing speed and base quality we determined the total time taken to sequence the template and complement bases per unit time per active channel. This provides a measure of the true mean rate that sequences were translocating through the pores. By incorporating the time for which active pores were not sequencing, an effective sequencing rate could be calculated. For a typical experiment, P1a-Lab2-R2, template and complement sequences were produced at a declining rate over the course of 24h. For both metrics, the rate at which template sequences translocate through the pore decreases more rapidly than the complement sequences ( Figure S5A). Plotting the average occupancy rate of pores over time, alongside the number of active channels over time, demonstrates that active pores continued sequencing at similar rates until they become inactive, which happened at a relatively uniform rate during an experiment ( Figure S5B). Thus, further investigation of how base quality ( Figure 8), read accuracy ( Figure 10) and the speed at which the DNA translocates through the pore ( Figure S5) over time may suggest strategies for improving base-calling.

### Proportion of 2D pass and fail reads

**Figure 11.**
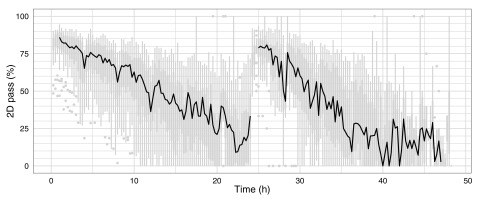
Percentage of 2D pass reads produced over time. Boxplots showing the proportion of 2D pass reads started in each 15 minute interval were plotted for the 20 experiments (grey), and the median values connected with a black line.

A base-called FAST5 file is classified as ‘fail’ if: (i) base-calling failed; (ii) no 2D base-calls were inferred; or (iii) the 2D base-called read had a mean quality score ≤ 9. All other reads are classified as ‘pass’ and can be considered the ‘high-quality’ reads from the experiment. Although there was substantial variability in the proportion of 2D pass reads produced during the experiments, there was a clear decrease in median percentage of 2D pass reads from 85% to 20% over the course of the first 21h of the experiment ( Figure 11). The drops in 2D pass yield coincide with the 4h bias-voltage adjustments ( Figure 11), suggesting the reads produced during these transition periods do not have correctly calibrated base qualities.

### Miscall, insertion and deletion rates of 1D and 2D base-calls

The median total error of all 2D reads was 12% ( Figure 10C, Figure S8A), with miscalls, insertions and deletions contributing 3%, 4% and 5%, respectively ( Figure 10C). The 2D pass reads had a slightly lower total error of 10.5% ( Figure 10A) and the 2D fail reads a much higher value of 20.7% ( Figure 10A), based on the best alignment strategy attempted, of BWA-MEM followed by EM correction by marginAlign. The error estimated from alignments with BWA-MEM alone were significantly higher: a median total error of 15% for all 2D reads ( Figure 10B), 11.6% for 2D pass reads and 22.6% for 2D fail reads ( Figure 10A).

The application of a better alignment algorithm, in this case the EM correction implemented in marginAlign, had the effect of decreasing miscalls at the expense of a slight increase in insertions and a small increase in deletions, with the net decrease in the total error of 1.9% for 2D fail base-calls and 1.1% for 2D pass base-calls ( Figure 10A). During an experiment, the total error inferred from BWA-MEM alignments increased during the first 24h of the experiment, dropped at the 24h re-mux and library reload, then increased again until the experiment was terminated ( Figure 10). Use of a better alignment algorithm not only reduced the miscall, insertion, and deletion rates, but resulted in a more uniform profile of each error type during an experiment, and in particular, reduced the rate of increase of deletions during an experiment ( Figure 10C). The 4h periodic effect observed previously in the base quality plots is also clearly evident in the error plots ( Figure 10B).

**Figure S6.**
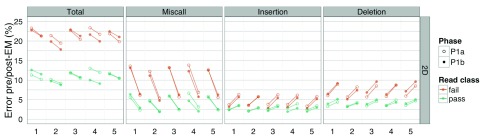
Error estimates for target 2D base-calls from each phase. The pre- and post-EM percentage error for BWA-MEM alignments of target 2D base-calls, grouped by phase. There was little difference between the error rate of Phase 1a and 1b experiments.

**Figure S7.**
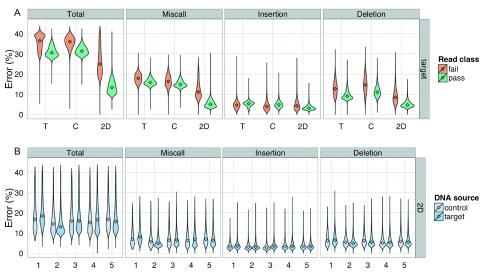
Variation in base-call error across read types and DNA source. The total error, and the contribution of miscalls, insertions and deletions for 2D base-calls of target reads for ( **A**) template, complement and 2D base-calls split by the pass and fail classification, and ( **B**) samples from the target or control DNA, grouped by laboratory. The percentage error was estimated from BWA-MEM alignments without EM correction, and thus, higher than the corresponding values in Figure 10. However, these values are sufficient to show that the error rate of the 1D template and complement base-calls are similar, and about twice that of the 2D base-calls. And the error of the base-calls from pass reads are always lower than for the fail reads of the same read type. Similarly, the error estimates were similar for target and control base-calls across all laboratories.

**Figure S8.**
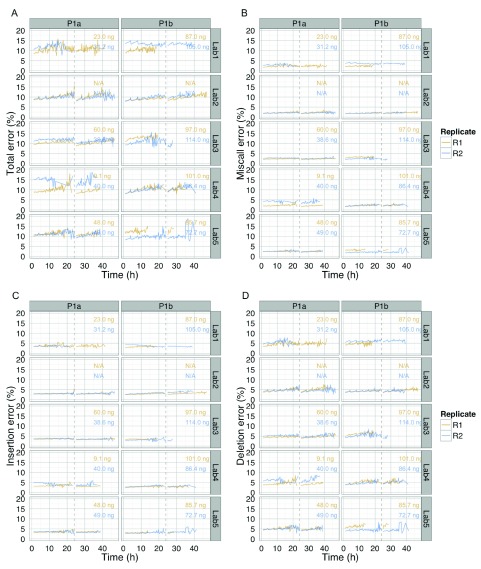
Variation in miscall, insertion and deletion error over time for 2D base-calls. These data are the same as shown in Figure 10C, inferred from BWA-MEM alignments followed by EM correction, but separated by phase and lab to more clearly show the trends over time for each experiment. The total percentage error of individual reads, and the miscall, insertion and deletion components, were almost constant over time, but interrupted by an increase in error for reads that were sequenced during the 4h bias-voltage adjustments.

Error rates inferred from the use of the BWA-MEM and LAST as the initial aligner were very similar; therefore, only the values based on BWA-MEM are described. The error estimates from BWA-MEM, pre- and post-EM alignment, were very similar for experimented from Phase 1a and 1b ( Figure S6). Error estimates inferred from BWA-MEM alignments without EM correction showed that the error rate of the 1D template and complement base-calls were similar, and about twice that of the 2D base-calls; and the error of the base-calls from pass reads were always lower than for the fail reads of the same read type ( Figure S7A). Similarly, the error estimates were similar for target and control base-calls across all laboratories ( Figure S7B). The total percentage error of individual reads, and the miscall, insertion and deletion components, were almost constant over time, but interrupted by an increase in error for reads that were sequenced during the 4h bias-voltage adjustments ( Figure S8).

### Correlation between base quality and read accuracy

**Figure 12.**
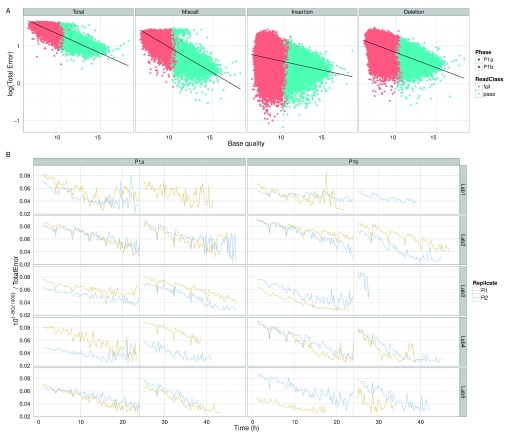
Relationship between accuracy and base quality. ( **A**) The percentage error (on a log scale) plotted against the mean base quality of each 2D read. Reads from the Phase 1a and 1b experiments are distinguished by shape and the pass and fail read types by colour. The relationship between total error, and the miscall, insertion and deletion components, are shown separately. The linear regression line demonstrates that base quality and error are related by an exponential function. ( **B**) The variation in 10 ^(-Q/1000)^/TotalError over time for each experiment. Although the value should be constant for all reads, the value declines over time. The characteristic unusual values occurring every 4h suggest that base quality is not as well correlated with accuracy for reads that were being sequenced during a bias-voltage adjustment.

According to the metadata in the FAST5 data files ( Table S2), the base quality Q is related to the probability of error p by the Phred scale formula Q = -1000log _10_(1-p). The linear relationship between the logarithm of percentage error and the mean base quality of 2D reads mapped with BWA-MEM confirms this relationship ( Figure 12A), thus demonstrating that base quality is correlated with the accuracy of base-calls and can be used to filter reads of an unknown genome to the accuracy required for a particular analysis. We suspect the decrease of 10 ^(-Q/1000)^/TotalError over time, a value that should be the same for every read, was the result of decreasing mean read base qualities during an experiment and the 4h dips in the signal were due to the miscalculation of the mean base quality of reads that were being sequenced during a bias-voltage adjustment ( Figure 12B).

### Proportion of base-calls in long reads

One attribute that distinguishes nanopore sequencing from many next generation technologies is the possibility of acquiring base-calls that are over 10,000 bases long. Typically, 7.6%, 4.0%, 4.4%, and 3.6% of the reads had over 10,000 bases in the template, complement, 2D, and 2D ‘pass’ base-calls ( Figure S1A). Similarly, 50% of reads had a length of at least 5,500, 5,600, 6,000 and 6,300 bases for the template, complement, 2D, and 2D ‘pass’ base-calls ( Figure S1B). Generally, 5% of the reads had a length of at least 14.5, 13.0, 13.5 and 13.6 × 10 ^3^ bases for the template, complement, 2D, and 2D ‘pass’ base-calls ( Figure S1B). The longest template, complement, 2D, and 2D ‘pass’ base-calls observed in this study were 291.6, 300.5, 59.7 and 59.7 × 10 ^3^ bases, respectively.

### Accuracy of consensus sequences

The median theoretical fold coverage of the target *E. coli* genome achieved by the 20 experiments was 25 for 2D reads (min=5.2, Q1=16.3, median=24.9, mean=29.0, Q3=36.5, max=78.5) and 16 if restricted to 2D ‘pass’ reads (min=1.7, Q1=11.3, median=15.9, mean=20.3, Q3=27.0, max=47.9). When the theoretical fold coverage of all 2D base-calls or just the 2D ‘pass’ base-calls was at least 20, 99.9% of the sites were called accurately by the majority consensus. A theoretical fold coverage of at least 60 was required to call 99.99% of the reference sites accurately from the majority consensus.

### GC content 2D base-calls

**Figure 13.**
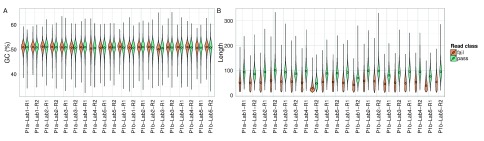
GC content and best perfect subsequences. The distribution of ( **A**) read GC content as a percentage; and ( **B**) the length of the best perfect subsequences of target 2D pass and fail base-calls from each experiment.

The GC content of 2D base-calls of the *E. coli* sample were very close to the actual value of 50.8% for all experiments, with some variation between the pass and fail base-calls ( Figure 13A).

### Sequence motifs with lower accuracy

The under-represented 5-mers for the 2D base-calls of the target and control samples suggest the nanopore sequencing technology has difficulty sequencing homopolymers ( Table S9). Homopolymers and repeated bases were also prominent in the table of over-represented 5-mers, but the mechanism producing this phenomenon is not clear ( Table S9).

### Longest perfect subsequence in 2D base-calls

The length of the longest perfect subsequence in the base-calls of each read is a measure of sequencing accuracy. The median length in 2D base-calls of the target sample was 50 and 90 for the fail and pass base-calls, respectively, across all experiments except P1a-Lab4-R2, which may have been run with a higher concentration of fuel mix ( Figure 13B). However, the distribution for all experiments had a long tail, the longest exceeding 300 consecutive, perfect bases ( Figure 13B).

## Discussion

The overall objective of MARC is to provide a definitive description of the Oxford Nanopore Technologies sequencing platform through a flexible publication strategy that accommodates the rapid pace of nanopore sequencing technology development. In this first phase of the MARC collaboration, we generated 20 datasets at five laboratories on different continents for the same *E. coli* bacterial strain, with sufficient lab replicates to be able to quantify the data yield, quality, accuracy and reproducibility that can be expected from the MinION, flow cells, chemistry, software and protocols available in April 2015. We demonstrated that there was considerable variability in the quality of flow cells, but all flow cells that had a high number of active pores when they arrived at their destination laboratory produced data of comparable yield, quality and accuracy. This dataset, the largest replicate sequencing effort of its kind on nanopore sequencing to date, is published here to allow continued independent investigation by the broader scientific community and enable more rapid development of algorithms and software for these data.

The MARC Phase 1 experiments were designed to provide benchmark data that explored the relative contributions of instrument, flow cell, laboratory and user to the variation in MinION system performance observed by the MAP community. The experiments in this study ( Table S6) followed a standard protocol based on that recommended by Oxford Nanopore at the time of the study, with clear choices made for the procedure to be followed when optional or open-ended steps existed. The protocol that we followed in this study ( File S1 MARC protocol) was based on the standard SQK-MAP005 protocol provided by Oxford Nanopore (version MN005_1124_revC_02Mar2015, last modified 10 June 2015), the only amendment being the use of 12 µl of library in Phase 1b and annotations to make the protocol clearer.

The large number of replicates allowed us to make generalisations about the data yield and quality. Utilizing version R7.3 flow cells and SQK-MAP005 chemistry, a typical experiment yielded 115 million 2D bases in ~20,000 reads with a median protocol-specific shearing length of 6,500 bases and mean base quality of 11.2. When the 8 Kb shearing protocol was used, approximately 4.5% of the 2D reads had a length of at least 10,000 bases, with some having a length of over 50,000 bases. Up to 10% of the reads of an experiment were from the DNA CS control added during library preparation. About 32% of the reads from an experiment result in 2D reads from the target genome. The accuracy of base-calls decreases during the course of an experiment. However, the total error of individual 2D base-calls was ~12%, with miscalls, insertions and deletions contributing ~3%, ~4% and ~5%, respectively. A single experiment yielded sufficient 2D bases for ~25-fold coverage of the target *E. coli* genome. When restricted to 2D ‘pass’ reads, the yield decreased to ~12,000 reads containing 75 million bases with a read length distributed centred around 6,700 bases and a mean base quality of 11.9. A 2D base yield corresponding to at least 20-fold coverage of the target genome was required to correctly call 99.90% of the 4.6 Mb *E. coli* genome, and 60-fold coverage to correctly call 99.99% of the genome, from the majority consensus of mapped reads.

Although the MARC standard protocol was documented in great detail, the quantity and quality of the output data varied due to many steps being sensitive to the quality of the materials and reagents used, stochastic variation in the application of the steps, accidental deviations from the protocol, and unexpected computer failures during a sequencing run. A large component of variability in MinION data quality was contingent on lab-specific behaviour. Although a number of minor deviations from the standard MARC protocol occurred, we found that the wet-lab method variations (e.g., DNA mass used to prepare a library, sheared length of DNA or the volume of library loaded on a flow cell) and occasional failures of computer software or hardware affected reproducibility but had minimal effects on data quality. The one notable exception was the amount of fuel mix, where a higher concentration of fuel mix loaded at the start of run P1a-Lab4-R2 was the most plausible explanation for the unusually high sequencing rate, shorter reads and poorer base qualities observed. According to Oxford Nanopore, a ‘fast mode’ enhancement will soon become available, including fine tuning of the event detection parameters to ensure that long read lengths are maintained upon addition of more fuel mix to increase speed.

The MinKNOW program, that uses sequencing protocol scripts to control the MinION device, was regularly upgraded during the study, as was the Metrichor agent that performed base-calling. In both instances, sequencing related parameters were similar during the period of our investigation. However, local forced restarts of the scripts were found to be the largest source of variation among the 20 runs, resulting in extreme variation in the length of the sequencing run, event yield and the event generation profiles. Restarts alter the specific pores being used for sequencing via mux selection and also disrupts a very prescribed bias-voltage profile required for an ideal ‘fresh’ flow cell to operate optimally through a 48h sequencing run. Alteration away from the ‘standard’ experimental conditions can therefore have a large impact on the performance of a flow cell, both positive and negative depending on parameters used, and confounds comparative analysis.

The performance of the MinION device itself was consistent. Each experiment ran at a characteristic temperature within an acceptable range that did not fluctuate during an experiment and no experiments experienced failures due to problems with the device. Although GC biases may be hard to detect through the sequencing of an *E. coli* strain with a mean GC content close to 50%, we did not observe a genome-wide GC bias in the 2D reads produced by this platform. Neither longer target nor shorter control library molecules were sequenced preferentially during the experiments, and the accuracy of target and control base-calls was very similar.

The most important determinant of data yield was the initial number of active pores in the flow cell. On delivery, ~60% of all the pores on the flow cell were usable and the best flow cells had ~95% and 80% active pores in the g1 and g2 well-groups at the commencement of an experiment. Active pores were sequencing for ~90% of their active time, with a uniform idle period between library molecules suggesting pores have consistent performance until they become inactive. The first hour of a run is generally predictive of total run yield. Flow cells that commenced sequencing with at least 400 of the maximum of 512 well-group g1 pores yielded optimal event yield profiles from high quality libraries.

The similarity of the 2D base quality profiles from the same lab suggest the base quality of an experiment may be dependent on the characteristic human or equipment-related sequencing conditions in a laboratory. But it is also possible that it may be due to the shipping procedure to that location. Thus, the reason for the decrease in base-call accuracy during an experiment is still not fully understood, but the large number of replicate experiments in this study, carried out in five laboratories on different continents, is the best available resource for exploring the possible mechanisms. The characteristic trend observed for all metrics of data quality produced by the current group of pores was a steady decrease over time, punctuated by a fluctuation every 4h coinciding with the pre-set bias-voltage adjustment. We hypothesize that variations in sequencing rate (measured in bases per second) were caused by decreasing flow cell performance over time that is not accounted for in the base-calling models. The adjustments in bias voltage every 4h appear to mitigate some of these effects, but the frequency of these adjustments do not track the changing state of the flow cell closely enough to result in uniform data quality during an entire experiment. This suggests the pre-programmed bias-voltage adjustments have been optimized for the library preparation protocol recommended for that flow cell chemistry, and the particular volumes of library and fuel expected during the sequencing run. As such, software or protocols that could maintain synchrony between these two aspects of the sequencing process may significantly improve the overall performance of the technology and confirm that re-calling bases of older experiments with new software is probably not advisable.

The addition of more library and fuel mix coincided with the switch from the use of the g1 to g2 pores, so it was not possible to tell which of the two factors was responsible for any changes in data yield or quality, or whether the lower overall performance in the second 24h period of the experiment may have been due to degradation of the DNA, adapters or motor proteins during 24h of storage. However, the increase in read production rate ( Figure 4), and quality after the 24h mux switch suggest ‘fresh’ pores and/or sample produce higher quality data ( Figure 8). Given that the base-calling algorithm is tuned to use normalized current profiles, ‘mid’-read bias-voltage changes would compromise this process and we hypothesize it causes a disproportionate decrease in the quality of the base-calls for a short transition period until complete reads are produced under the same ionic driving force. The similarity of the 2D base quality profiles from the same lab suggest the base quality of an experiment may be dependent on the characteristic human or equipment-related sequencing conditions in a laboratory ( Figure 9). The two Phase 1a and 1b replicate experiments performed in Lab 2 and Lab 5 were run concurrently on different MinIONs while all other laboratories performed the replicate experiments sequentially ( Table S8). The 2D plots for replicate experiments from these two labs are the only pairs of experiments that have a different rate of decrease, which suggests the MinION itself has some influence on the decrease in base quality over time ( Figure 9A).

Finding standard metrics for assessing the error of the long single-molecule reads was a challenge. Alignment-free approaches based on k-mer frequencies have lower accuracy for homopolymeric regions or those with a low or high GC content ( Laehnemann *et al*., 2015). If a platform is capable of sequencing any DNA sequence, all possible 5-mers in the DNA should be proportionally represented in the data when counts are normalized for the distribution of all 5-mers in the genome. Thus, the most under-represented and over-represented 5-mers in the base-calls from the MinION may suggest limitations or biases of the nanopore sequencing process. Conversely, using alignment-based approaches, we have observed that stretches of 90 perfect bases in 2D ‘pass’ reads and 50 bases in 2D ‘fail’ reads were typical ( Table S9), and that stretches of over 300 perfect bases were possible from the SQK-MAP005 chemistry. The accuracy (or error) values quoted in other studies have been difficult to compare because: (i) the precise values quoted are sensitive to the alignment method used to compare reads to the reference; (ii) there is a significant difference in the quality of the 2D ‘fail’ and ‘pass’ reads; and (iii) basing values on reads from both target and control DNA may affect the values if they have different GC contents. Quoting the percent identity of a read with respect to a reference can be misleading because an increase in the percent identity can be induced by a decreased rate of insertions or deletions. We found that the total error of 2D ‘fail’ and ‘pass’ reads was 23% and 12%, respectively, using the nanopore-tuned parameters for BWA-MEM, but re-alignment using an EM technique reduced the error to 21% and 12%, respectively. In fact, we expected error rates to differ between phases (due to different chemistries) and samples (due to different types of input DNA), but instead the only observed error rate differences were between the type of read (template/complement/2D) and whether or not it had been classified as pass or fail. Although the error of individual MinION reads is high compared to those from the more established short-read technologies, it has been demonstrated that these data are of sufficiently high-quality to infer full-length *de novo* assembly of the *E. coli*, Influenza virus, and *Saccharomyces cerevisiae* genomes ( Goodwin *et al*., 2015; Loman *et al*., 2015; Quick *et al*., 2014; Wang *et al*., 2015).

Although reported, the 1D reads were not fully explored and it is acknowledged that discounting these data likely underestimates error and reduces usable data. If there are regions of the target genome that only have coverage by template base-calls, the demonstrated correlation of mean base quality and accuracy could be used to select the more accurate 1D reads that exceed an appropriate base quality threshold.

The observations from this study suggest there are many ways in which the performance of the MinION platform could be improved. Clearer protocol steps, that describe software steps, could reduce mistakes and computer issues. Methods that deliver longer, intact library molecules to the flow cell would have a large impact on the length distribution of the resulting base-calls. Improved run scripts, that utilize the best available pore for each channel rather than relying on pre-defined well-groups, could dramatically increase data yield and quality. Improvements in base-call accuracy through finer-grained regulation of bias-voltage adjustments may be possible, but these would need to be accompanied by more accurate mean base qualities for reads that span voltage transitions. Yield of the target sequence could be improved by reducing the volume of the control sample in the library. Investigation of motifs that have no coverage in the 2D base-calls may suggest a means of alleviating these limitations. Development of base-calling algorithms that take into account the methylation profile of the target DNA could reduce the regions of the genome that are consistently unrecovered by the current technology. The lifetime of a flow cell is not limited to 48h, and this study demonstrates that significant amounts of additional data can be generated if sufficient active pores remain.

The data generated in this study are intended as a snapshot of the state of the MinION technology in April 2015. There are many other analyses that could have been presented here, but to release the datasets to the wider community rapidly, we have deliberately performed only preliminary analyses and hope the release of these datasets will inspire the development of software based on new algorithms that specifically address the unique properties of data from the MinION platform. We hope that more analyses will be performed on this dataset both by MARC members and others. During Phase 1 of the MARC collaboration, new minor versions of the flow cell chemistry and software were released, and the first ‘field’ runs of the new MinION Mk1 device with the new flow cells using SQK–MAP006 reagents and updated base-calling software based on 6-mers commenced in late September 2015. To provide a link between the data presented in this study and the MinION Mk1 data, MARC will conduct ‘bridging experiments’ to evaluate the differences in the data yield and accuracy and error profile, before embarking on the MARC Phase 2 experiments to identify protocol changes that improve the performance and extend potential applications of the platform.

## Data availability

The data referenced by this article are under copyright with the following copyright statement: Copyright: © 2015 Ip CLC et al.

The raw and aligned nanopore reads, and files of statistics for each of the 20 experiments ( Table S10) are available from the European Nucleotide Archive project PRJEB11008 ( http://www.ebi.ac.uk/ena/data/view/PRJEB11008).
