# Supplementary material for: MinION Analysis and Reference Consortium: Phase 1 data release and analysis
Source: F1000Res. 2015 Oct 15;4:1075. [Version 1] doi: 10.12688/f1000research.7201.1 (PMC4722697; doi:10.12688/f1000research.7201.1)
Supplement: Supplementary file 1 [file f1000research-4-7757-s0000.tgz › 704d5301-1c0c-47fe-aaf5-3a79fca1628d.docx]

**Protocol for MARC Phase 1**

**Reagents**

1. Qubit dsDNA HS Assay Kit (Invitrogen cat. no. Q32854)
2. Genomic DNA ScreenTape and Reagents (Agilent technologies, cat. no. 5067-5365, 5067-5366)
3. LB broth
4. QIAGEN Genomic-tip 20/G (cat. no. 10223)
5. QIAGEN Genomic DNA Buffer Set (cat. no. 19060): Buffers B1, QBT, QC and QF
6. Lysozyme (Sigma cat. no. L6876 )
7. RNase A (QIAGEN cat no. 19101)
8. QIAGEN Proteinase K (cat. no. 19131)
9. Isopropanol
10. 10 mM Tris-Cl, pH 8.5
11. Covaris g-TUBE (cat. no. 520079)
12. Agencourt AMPure XP beads (Beckman Coulter Inc., cat. no. A63880)
13. NEB “PreCR Repair Mix” (New England BioLabs, cat. no. M0309S)
14. dNTPs (New England BioLabs, cat.no. N0446S)
15. NEBNext End Repair module (New England BioLabs, cat. no. E6050S)
16. NEBNext dA-Tailing module (New England BioLabs, cat. no. E6053S)
17. Blunt/TA Ligase Master Mix (New England BioLabs, cat. no. M0367S)
18. 80% Ethanol
19. Dynabeads His-Tag Isolation and Pulldown (Life Technologies, cat.no. 10103D)
20. MinION device and the USB3 cable
21. R7.3 flow cell
22. Genomic DNA Sequencing Kit SQK–MAP005 (Oxford Nanopore Technologies)

**Day 0 : Collating reagents and clearing the laptop hard disk**

1. Collate all the above reagents, in addition to the MinION device and the USB3 cable to connect the device to the laptop.
2. Ensure sufficient hard drive space is available on your laptop and no unnecessary software is running.

**Day 1 : Sample culture (overnight)**

1. Using a sterile P200 pipette tip, take 1 bead and put in 10 ml LB broth.
2. Grow the bacteria at 37°C overnight.

**Day 2 : DNA extraction (1 day)**

1. Measure bacterial growth by mean of a spectrophotometer and make sure to use no more than 4.5 x 10^9^ cells for each Genomic-tip.
2. Pellet bacteria from an appropriate volume of culture by centrifugation at 3000–5000 x g for 5–10 minutes.
3. Discard the supernatant, ensuring that all the liquid is completely removed.
4. Meanwhile prepare a stock solution of Lysozyme by dissolving lysozyme in distilled water to a concentration of 100 mg/ml;

**NOTE: store lysozyme at -20 °C for future use in small aliquots to avoid multiple cycles of freeze/thaw.**

1. For each extraction prep, add 2 µl of RNase A solution (100 mg/ml) to a 1 ml aliquot of Buffer B1.
2. Resuspend the bacteria pellet in 1 ml of Buffer B1 (with RNase A) by vortexing at top speed; ensure that the pellet is fully dissolved.
3. Add 20 µl of lysozyme stock solution (100 mg/ml), and 45 µl of QIAGEN Proteinase K stock solution. Incubate at 37°C for at least 30 min.
4. Add 350 µl of Buffer B2, and mix by inverting the tube several times. Incubate at 50°C for 30 min.
5. Equilibrate a QIAGEN Genomic-tip 20/G with 1 ml of Buffer QBT, and allow the QIAGEN Genomic-tip to empty by gravity flow.
6. Vortex the sample for 10 seconds at maximum speed and apply it to the equilibrated QIAGEN Genomic-tip. Allow it to enter the resin by gravity flow.
7. Wash the QIAGEN Genomic-tip with 3 x 1 ml of Buffer QC.
8. Elute the genomic DNA with 2 x 1 ml of Buffer QF.
9. Precipitate the DNA by adding 1.4 ml (0.7 volumes) of room temperature 100% isopropanol to the eluted DNA.
10. Precipitate the DNA by inverting the tube 10 to 20 times, and spool the DNA using a glass rod.
11. Immediately transfer the spooled DNA to a microcentrifuge tube containing 0.1–2 ml of 10 mM Tris-Cl, pH 8.5.
12. Gently dissolve the DNA overnight on a shaker at room temperature.

**Day 3 : Library preparation and start of experiment (~4 hours)**

1. Determine the concentration of the extracted DNA by Qubit dsDNA HS Assay and the integrity on a Genomic DNA ScreenTape (Tapestation).

**Fragmentation and PreCR**

1. Shear 1 µg of genomic DNA in a Covaris g-TUBE in a total volume of 80 µl by spinning at 3,381 x g for 1 minute.
2. Perform PreCR treatment of fragmented DNA by gently mixing the following reagents:

80 µl Fragmented DNA

6 µl Nuclease-free water

10 µl 10x Thermopol buffer

1 µl 100x NAD+

1 µl 10 mM dNTPs

2 µl PreCR repair mix

1. Spin down, and incubate the reaction at 37 °C for 30 minutes.
2. Clean up the reaction products with 1x Agencourt AMPure XP (100µl) beads:
   - Add the AMPure XP to DNA and mix thoroughly by pipetting.
   - Incubate for 5 minutes at room temperature.
   - Transfer tubes to magnet and incubate for 8 minutes.
   - Aspirate off supernatant, once it is clear and colourless.
   - Do not transfer any beads in the supernatant. If this is a risk, leave <5µl supernatant behind with the beads.
   - Keeping the tube on magnet, wash beads with 200µl 80% ethanol without resuspending. Leave for 30 seconds.
   - Remove and discard the supernatant. Repeat wash with ethanol.
   - Ensure removal of all ethanol.
   - Air dry beads until the well looks dry and the beads are starting to crack.
3. Resuspend beads in 82µl 10 mM Tris-HCl pH8.5.
4. Return tubes to magnet for 2 minutes.
5. Transfer eluant to clean tube.
6. Determine the concentration of the extracted DNA by Qubit dsDNA HS Assay and the fragment size on a Genomic DNA ScreenTape (Tapestation).

**End repair**

1. Add 5 µl of DNA CS to the 80 µl of fragmented DNA.
2. Perform end-repair by adding:

10 µl NEBNext End Repair Reaction Buffer (10X)

5 µl NEBNext End Repair Enzyme Mix

1. Incubate 30 minutes at 20°C.
2. Clean up with 1x Agencourt AMPure XP (100µl) beads by volume afterwards (see 26 for details).
3. Resuspend beads in 27µl 10 mM Tris-HCl pH8.5.
4. Return tubes to magnet for 2 minutes.
5. Transfer eluant to clean tube.
6. Determine the concentration of the end-repaired DNA by Qubit dsDNA HS Assay.

**dA-tailing**

1. Perform dA-tailing using NEBNext dA-Tailing Module by adding to 25 µl of DNA:

3 µl NEBNext dA-Tailing Reaction Buffer

2 µl Klenow Fragment (3´→ 5´ exo–)

1. Incubate 30 minutes at 37°C
2. Clean up with 1x Agencourt AMPure XP (30µl) beads by volume afterwards (see 26 for details)
3. Resuspend beads in 32µl 10 mM Tris-HCl pH8.5.
4. Return tubes to magnet for 2 minutes.
5. Transfer eluant to clean tube.
6. Determine the concentration of the A-tailed DNA by Qubit dsDNA HS Assay.

**Ligation**

1. Assemble the following reagents in the order given below, in a Protein LoBind 1.5 ml Eppendorf tube:

30 µl dA-tailed DNA

10 µl Adapter Mix

10 µl HP Adapter

50 µl Blunt/TA Ligase Master Mix

1. Mix by pipetting between each sequential addition:

**CRITICAL STEP: after adding the adapters, only mix by pipetting and avoid contacting the solution with parts of the tube that are above the level of the reaction mix.**

1. Briefly spin down in a microfuge and incubate for 10 minutes at room temperature.

**Start of MinKNOW and Platform QC**

1. Attach the flow cell to the MinION sequencer, ensuring a good contact between the heatsink on the underside of the flow cell and the MinION device.
2. Position the sequencer on a flat surface, away from the laptop, where it cannot be bumped accidentally while the experiment is being monitored on the laptop.

**NOTE: the output of a sequencing run on the MinION is highly dependent on the number of available pores in the flow cell. Before a new sequencing run, it is recommended to run a Platform QC of the new flow cell to assess that number.**

1. To start the MinKNOW software that controls the MinION, open the MinKNOW Control Panel and click the “Start” button.
2. In the MinKNOW native client window, record the flow cell ID and enter a name for the QC run
3. Select the MAP_Platform_QC.py script to start the QC of the flow cell.
4. At the end of the Platform QC, record the total number of active channels and the number of active pores in each of the g1, g2, g3 and g4 well groups.

**NOTE: A number of active channels above 400 in g1 is considered acceptable.**

**His-tagging**

1. Resuspend His-tag beads by vortexing for 30 seconds.
2. Take 450 µl of the 2x Bead Binding Buffer (2x BBB) and add 450 µl nuclease-free water.
3. Mix by inverting 10 times and briefly spin down in a microfuge. This 1x Bead Binding Buffer (1x BBB) is required for preparing the beads and in the subsequent purification of adapter-ligated DNA.
4. Take 10 µl of His-beads and transfer to a clean 1.5ml Protein LoBind tube.
5. Wash the beads by resuspending in 200 µl of 1x BBB.
6. Place the tube on a magnet and allow the beads to pellet before aspirating off the supernatant.
7. Repeat the wash step (58-59).
8. Resuspend the washed and pelleted beads in 100 µl of the 2x BBB: these are the “washed beads” required in the subsequent purification.
9. To the adapter-ligated DNA add 1x (100 µl) of “washed beads” and carefully mix by pipetting
10. Incubate at room temperature for 5 minutes.
11. Place on a magnetic rack, allow the beads to pellet and remove supernatant.
12. Rinse the pelleted beads in 200 µl 1x BBB.
13. Remove the 1x BBB (do not disturb pellet).
14. Rinse again the pelleted beads in 200 µl 1x BBB
15. Remove the 1x BBB (do not disturb pellet).
16. With the lid of the tube closed, briefly spin in a microfuge, sufficient to drain excess wash buffer from the beads.

**CRITICAL STEP: remove any residual**  Bead Binding Buffer **with the pipette only and not by evaporation, as the salt in the buffer might affect the motor proteins**

1. Replace the tube on the magnet to pellet the beads and leave for approximately 1–2 minutes, still with the lid of the tube closed.
2. Aspirate off any remaining 1x BBB.
3. Resuspend the pelleted beads by gentle pipetting in 25 µl of Elution Buffer, adding the buffer close to the beads avoiding “washing” any residual buffer off the sides of the tube as much as possible and ensuring that unnecessary washing of residual 1x BBB from the sides of the tube is avoided.
4. Wait for 10 minutes before pelleting on a magnet rack.
5. Remove eluate which contains the library: this library is called the Pre-sequencing Mix.
6. Determine the concentration of the Pre-sequencing Mix by Qubit.

**Priming of the flowcell**

**NOTE: Only use a P1000 pipette to load the flow cell**

1. Prepare a mixture of 6.5 µl Fuel Mix, 162.5 µl Running Buffer and 156 µl nuclease-free water in an Eppendorf tube. Mix thoroughly by vortexing and spin down the contents of the tube.
2. Inspect the sample port, the inlet channel and the active area of the flow cell to make sure the buffer is continuous and there are no air bubbles present.
3. If an air bubble is present, gently draw back the air bubble into the tip. Keep drawing back until there is a continuous flow of buffer into the tip. There is excess buffer in the flow cell to allow this process but beware of drawing back too much.
4. Load 150 µl of this mixture into the flow cell and wait for at least 10 minutes.
5. Load a further 150 µl of the mixture into the flow cell and wait for at least 10 minutes.

**Loading the library onto the flowcell**

1. Take 6 µl of the Pre-sequencing Mix, add 75µl Running Buffer and 66 µl nuclease-free water.
2. Add 3 µl Fuel Mix (Fuel Mix must be added last) and mix by pipetting
3. Load the 150 µl mix to the flow cell.
4. Store Pre-sequencing Mix, Running Buffer and Fuel Mix at 4°C.

**Start the sequencing experiment**

1. In the MinKNOW native client enter the Run Name, which will form the basis of the output data file names.
2. Select the the MAP_48Hr_Sequencing_Run.py protocol script to start the sequencing experiment.

**NOTE: At the beginning of a sequencing run, the software will perform a new flow cell QC and allocate wells to well-groups that will be used during the 48 hour run.**

1. Record the total number of active channels and the number of active pores in each of the g1, g2, g3 and g4 well groups.

**Start the Base-calling**

**NOTE: Before starting the base-calling, make sure enough space is available on the hard drive for both the base-called files and the reads files created by MinKNOW during the sequencing run. If space is limited, Metrichor could be started at the end of the run instead**

1. To start the base-calling, start the Metrichor Agent and proceed as follow:
   - Select “Configure Settings”.
   - Enter the API key, if required.
   - Under “Upload directory”, select the /reads folder created by MinKNOW after starting the sequencing protocol and containing the raw .fast5 files that need to be sent to the base-calling service by Metrichor.

**NOTE: the /reads folder will be created by MinKNOW in the location selected for writing files on the hard drive during the installation of the software**

- - Under “Download directory”, select the folder into which the files returned by Metrichor, containing the base-calls, will be saved. This could be the “default” /reads/downloads folder or a custom folder. Metrichor will create sub-folders called “pass” and “fail”, each containing the base-called FAST5 files, and a single file called “telemetry.log” in the “downloads” folder.
  - Select “Run a workflow” and from the workflows list, select the 2D base-calling.

**Day 4 : Sequencing (all day)**

**Reloading the library onto the flowcell**

1. After the MAP_48Hr_Sequencing_Run script has been running for 24h from the start of the run (i.e., when the MAP_48Hr_Sequencing_Run script was started), take 6 µl of the Pre-sequencing Mix, add 75µl Running Buffer and 66 µl nuclease-free water in a new Protein LoBind 1.5 ml Eppendorf tube.
2. Add 3 µl Fuel Mix and mix by pipetting
3. Load 150 µl of the mix to the flow cell.
4. Store Pre-sequencing Mix, Running Buffer and Fuel Mix at 4°C.
5. Check the hard disk usage. If there may be insufficient space for the next 24 hours of data collection, remove any non-essential files from the laptop and/or if the Metrichor base-calling service has been running, terminate Metrichor and move the existing base-called files to another computer.

**Day 5 : Termination of experiment**

1. At 48h since the start of the run, check the MinKNOW diagnostic screens to ensure that the sequencing protocol has completed.
2. If the Metrichor agent had been started and had been producing base-calls:
   - ensure that the Metrichor diagnostics show that the number of FAST5 files from the /reads folder that have been sent to the cloud-based service agrees with the number of .fast5 files that have been returned to the chosen “Downloads” folder.
   - Take screen shots of the Metrichor agent diagnostic screens, if required.
   - Close the Metrichor agent.
3. Take any screen shots of the MinKNOW Software Agent, if necessary.
4. Close the MinKNOW agent.
5. Move entire contents of the Run Folder to a separate disk storage area to enable downstream analysis of the data and make the laptop ready for the next MinION sequencing experiment.
6. Store flow cell until it can be returned to Oxford Nanopore Technologies.
