# Supplementary material for: MinION Analysis and Reference Consortium: Phase 1 data release and analysis
Source: F1000Res. 2015 Oct 15;4:1075. [Version 1] doi: 10.12688/f1000research.7201.1 (PMC4722697; doi:10.12688/f1000research.7201.1)
Supplement: Supplementary file 2 [file f1000research-4-7757-s0001.tgz › 0b8a73be-67ee-444e-813c-9635aee56840.docx]

**Glossary**

| **Term** | **Description** |
| --- | --- |
| ASIC | Application-Specific Integrated Circuit. The electronics embedded to the underside of the flow cell that interpret the data from the sensor in a well. |
| base-called read | A read containing either raw current measurements or events, and the base-calls inferred from the raw current measurements or events. |
| base-calls | The sequence of bases inferred for a read. Each base-called read may contain up to three sets of base-calls for; (i) the ‘1D’ template strand of the library molecule; (ii) the ‘1D’ complement strand of the library molecule; and/or (iii) the ‘2D’ base-calls inferred from the 1D information. |
| base quality | The quality score associated with each base of the template, complement of 2D base-calls. |
| batch id | The name of the ‘run number’ identifier allocated to the reads of each instance of the sequencing script. The ‘run number’ has the format 1234_5 and is part of the pre- and post-base-called read filename. |
| bulk data | The reads containing information on every ionic current measurement, rather than events. |
| bulk FAST5 file | A read file containing ‘raw data’, that is, all the ionic current measurements collected while the DNA passed through the nanopore. |
| channel | Electronic current monitoring stream from one of the 4 possible wells associated with each channel. The MinION has 512 channels, making it possible to sequence a maximum of 512 library molecules simultaneously. |
| complement base-calls | The base-calls inferred from the bases located after the hairpin adapter, corresponding to the complement strand of the double-stranded DNA used to construct the library molecule being sequenced. |
| complement read | The raw current measurements or the events collected while the complement part of the library molecule was passing through the nanopore. |
| event | An ionic current measurement usually corresponding to the sequencing of a base in a channel. It is specified by the current mean and variance (in picoAmps) and a duration (in units of 1/sampling_frequency). |
| failed read | A base-called read is classified as a ‘failed’ read, and the FAST5 file deposited into the ‘fail’ sub-folder of the directory into which Metrichor downloads base-called reads to the connected computer, if: (i) base-calling failed; (ii) no 2D read was produced; or (iii) the 2D read had a mean base quality score <= 9. |
| FAST5 file | A file in FAST5 format. |
| FAST5 format | An implementation of the HDF5 format used to store pre- and post- base-called nanopore data. Note that the internal format of the two types of FAST5 files are not the same (see SI Table 2). |
| flow cell | The chip that is attached to the MinION device. It contains a port into which the library can be loaded, a polymer membrane, and wells that contain (preferably one) containprotein nanopore. |
| hairpin adapter | Double-stranded DNA is made into a library molecule by ligating a leader-adapter to one end and a hairpin adapter (with a stem-loop structure) to the other. During sequencing, the presence of the hairpin adapter allows the complement part of the original double-stranded DNA to be sequenced and identified. |
| HDF5 format | A flexible, efficient, multi-purpose data format used in many different fields to store heterogeneous data in a hierarchy based on groups (analogous to directories) and datasets (analogous to files). A dataset may have a simple type like an integer or float, or be a complex type that is effectively a table built from simple types. |
| Metrichor™ | A company wholly owned by ONT that provides cloud-based analysis services, one of which is the nanopore base-calling. |
| Metrichor™ Agent | The software that sends reads to the ONT cloud-based basecaller hosted by Metrichor and returns the base-called reads to the local computer. |
| MinION™ | The device produced by Oxford Nanopore Technologies that can measure properties of single-stranded DNA as it passes through a nanopore. |
| MinKNOW™ | The software running on the computer connected to the MinION device, that controls the sequencing run and the detection of events, and saves the QC, diagnostic and read data to the connected computer. |
| mux | The process by which the quality of the pore in each of the 4 wells in a group are ranked and allocated, in order of best to poorest quality, to well-groups g1 through to g4. At the end of the process, each of the well-groups, referred to as g1 to g4, are associated with a maximum of 512 wells. For an experiment controlled by the standard 48Hr sequencing script, sequencing commences with the nanopores in the g1 wells, and at 24h, sequencing switches to the nanopores in the g2 wells. |
| mux scan | See re-mux. |
| nanopore | A protein pore, with a diameter measured in nanometers, through which DNA molecules can pass through. The nanopores are embedded in a membrane located in a well of the flow cell. Ideally, a single nanopore is embedded in each well. |
| Oxford Nanopore Technologies™ | The company that produces commercial sequencing devices based on nanopore the sequencing technology. Their MinION device sequences double-stranded DNA. |
| pass read | Any base-called read that was not classified as ‘failed’ (see ‘pass read’) has a classification of ‘pass’ and is deposited into the ‘pass’ sub-folder of the directory into which Metrichor downloads base-called reads to the connected computer |
| pore | See ‘nanopore’. |
| pore-switch | The transition by which the 512 channels use the active pores in wells of one group to a subsequent group. In the standard 48 hr sequencing protocol, a switch from the active pores in the g1 wells to the active pores in the g2 wells occurs at 24 hrs. |
| raw data | The ionic current measurements, measured several thousand times per second, by a sensor in a well. Raw data are found in ‘bulk FAST5 files’. Consecutive raw ionic current measurements that fluctuate around a particular value are merged into an event. |
| read | The data produced by the sequencing of a library molecule. A pre-basecalled read, produced by the MinION device and stored on the connected computer, contains metadata that documents how the read was acquired and either a sequence of every ionic current measurement made as the library molecule translocated through the pore, or the sequence of events consisting constructed by merging the raw ionic current measurements. |
| re-mux | Process by which active pores are identified and re-assigned to channels well-groups. |
| squiggle plot | A plot of the bulk or event current measurements over time. |
| template base-calls | The base-calls inferred from the first strand of the double-stranded sample DNA incorporated into the library molecule. |
| template read | The raw current measurements or the events collected while the template part of the library molecule was passing through the nanopore. |
| unclassified read | A read that cannot be classified as originating from DNA from a target or control sample because the read could not be aligned to the target or control reference(s). |
| well | A physical hole in the flow cell array containing a polymer membrane and zero or more protein nanopores. The current R7.3 flow cells contain 2,048 wells. Wells are grouped into sets of 4. The active well produces the data stream for the associated channel. |
| pore (group) switch | The transition by which the 512 channels receiving the data stream from the active pores in wells of one group switch to receiving the data stream from a subsequent group. In the standard 48h sequencing script, a switch from the active pores in the g1 wells to the active pores in the g2 wells occurs at 24h. |
