# Supplementary material for: MinION Analysis and Reference Consortium: Phase 1 data release and analysis
Source: F1000Res. 2015 Oct 15;4:1075. [Version 1] doi: 10.12688/f1000research.7201.1 (PMC4722697; doi:10.12688/f1000research.7201.1)
Supplement: Peer review history

# Peer review: Referee response for version 1

**DOI:** 10.5256/f1000research.7757.r10821

**Published:** 2015-11-23

**Authors:** Nicholas J. Loman

**Affiliations:** 1 Institute of Microbiology and Infection, University of Birmingham, Birmingham, UK

This manuscript produced by the MARC consortium is an impressively comprehensive study of run-level performance of the Oxford Nanopore MinION. The consortium have decided to focus on tightly defined parameters by sequencing a single bacterial strain, Escherichia coli K-12 MG1655, using a specific library preparation protocol and identical run parameters. Although some minor variability following the standard protocol between labs was observed, this is unlikely to affect the results.

It therefore provides the most extensive view of platform performance variability to date, focusing on a specific combination of library preparation chemistry (SQK-MAP-005) and flow cell type (R7.3). Its strengths are therefore in the emphasis on platform reproducibility. 

There are a few extra things that could have been done to make the results more akin to user experiences, such as not pre-filtering flow cells for those with >400 group 1 pores, but this is not a significant issue, but it would be nice to see a spread of performance of all tested flow cells. The performance reported is in line with our own experiences.

For me, the most interesting/useful elements of the paper were:

 

- the generally consistent results in terms of data quality achieved by different labs
- the extensive variability in throughput between flow cells which all give good QC (g1>400) values, suggesting that the QC stage is not a particularly reliable estimate of how well a flow cell will run
- Figure 1, which provides a useful summary figure (although please note caveats below)
- the close attention to detail to alignment methodologies
- the relationship between experiment run time, throughput and read quality, which suggest that a 48 hour workflow is not optimal, and pores should be remixed more frequently

A frustration is that the underlying reasons for the variability in performance, with the exception of operational issues, are not really explained and therefore these results do not really help users plan how to mitigate the variability. This is not the authors fault but remains an issue for those planning experiments that require a particular yield.

My major criticism is that the paper is over long and would have benefited from a good editor to try and reduce excessive verbiage. Sentences are often laborious and there is significant repetition throughout the manuscript. To pick on the first sentence of the manuscript: “the advent of a miniaturized DNA sequencing device with a high-throughput contextual sequencing capacity embodies the next generation of large scale sequencing tools”. This is fairly garbled. What is “contextual sequencing capacity”. And why does it “embody the next generation of large scale sequencing tools” ? A few hours with a proof reader would do wonders. Greater use of active voice would improve readability. But it is up to the authors to decide whether they want to spend more time revising the manuscript in this manner.

Generally I am happy with the manuscript to be approved as it is but I would suggest addressing a few technical points:

Figure 1. I could not figure out panels G or H easily. I could not figure out the relationship between the k-mers relating to each strand, they did not obviously seem to match up or be reverse complements of each other. Panel H I also cannot figure out how the 2D consensus sequence relates to the 1D reads. For example, why is there an insertion in the 2D sequence which does not have a corresponding alignment in the 1D?

In the section relating to the consensus sequences, the method seems to suggest that nanopolish was used to create a consensus sequence, using the reference sequence as the input alignment? I am not sure this is a particularly meaningful process. Accuracy measures like this (and any reference based alignment) are likely to be skewed by ‘reference attraction’, particularly given the alignment settings used - a pure *de novo *assembly may have been a more robust measurement. If that is too much work, the data could have been used (separately, or in combination) to polish the assembly from the nanopolish paper.

I wish the analysis had not made so much of the (ad hoc) separation of 2D pass and fail reads. In reality reads are in a continuum of quality and the pass filter is simply defined by the Metrichor workflow, which is presumably version dependent (and can be turned off). At the least a definition of the pass filter would be useful, from figure 12 it looks like it relates to a Q value of around 10. A detailed treatment of the “usability” of 2D pass versus 2D fail reads is missing, but this is probably out of scope in the paper.

One laboratory, Lab 3 reported high levels of Pseudomonas contamination. Although reagent contamination is a possibility, these very high levels are very unlikely to be due to vendor reagent contamination. Had this laboratory specifically handled P. putida in the recent past?

Minor nitpicking points:

Inconsistent use of trademarks e.g. MinION(TM), MinION in two contiguous sentences.

AGBT 2012 presentation has been posted in the F1000 channel and can be referenced.

“beta-testing” - is this formally defined or vernacular?

It is not clear how much of the description of the chemistry is informed guessing, based on company materials or from unpublished communications, it would be nice to clarify what is a definitive statement and what is speculative. Along that line, I did not realise that tethers are on both strand, is that definitely true? 

512 channels - this nomenclature is confusing, especially when compared to ‘wells’. Could this be clarified as to what a channel is?

I have read this submission. I believe that I have an appropriate level of expertise to confirm that it is of an acceptable scientific standard.

# Peer review: Referee response for version 1

**DOI:** 10.5256/f1000research.7757.r10824

**Published:** 2015-11-04

**Authors:** Michael Quail, Louise Aigrain

**Affiliations:** 1 Sequencing R&D, Wellcome Trust Sanger Institute, Cambridge, UK

The article supplied by the Minion Analysis and Reference Consortium is very thorough and a study that is worthy of indexation.

It is however outdated as the technology is moving so fast and few of the findings (aside from the description of alignment tools) are likely to be of much practical use to users. 

That said this is a useful study that is very worthy of indexation and will gain widespread interest.

I would recommend indexation (though it is already out there and has already been read by those interested) subject to the following revisions:

1. The authors introduce nanopore sequencing at the start of the introduction and should mention Nanopore sequencing technologies and approaches other than ONT e.g Noblegen, Genia, INanoBio, etc.
2. In paragraph 2 of the introduction the author say "a library is constructed from double-stranded DNA (dsDNA) with a protocol similar to that used for short-read, second-generation platforms" yet the library prep is more similar to that used by PacBio. Perhaps they should say "a library is constructed from double-stranded DNA (dsDNA) with a protocol similar to that used for other NGS platforms"
3. At the end of paragraph 2 of the introduction the authors say "Each channel provides data from one of the four wells at a time, the order of use defined by the allocation of wells to well-groups during an initial ‘mux scan’ (File S2 Glossary), allowing up to 512 independent DNA molecules to be sequenced simultaneously". I'm not sure that someone who hasn't used a MinION would understand what the MuxScan is and that it's an algorithm choosing towards which of the 4 surrounding pores each channel should point. The text should be modified to explain this better.
4. Paragraph 3 of introduction. Here the authors should make it clear that base calling doesn't take place on the computer connected to the MinION itself but can still be done while data is being acquired.
5. Paragraph 3 of introduction. The authors claim that "a single circular chromosome of 4.6 Mb that could be sequenced to sufficient depth in a single MinION run and a complete reference sequence is available". This is only true if a good flowcell with sufficient active pores is obtained. This should be made clear and the authors should declare how many flowcells they or ONT screened in order to get enough flowcells with sufficient active pores.
6. In figure 1 the authors should make it clear that the blue bar is the membrane.
7. At the start of page 6 in the section "Sequencer configuration and sequencing run conditions" the authors say that a minimum of 400 g1 channels was considered acceptable. This was actually a rare event with the minION versions the authors describe but is more consistent now. The authors should note that this threshold isn't always met and is one of the major factors in variability in data yield.
8. page 6, "Data Analyses section". The authors assume that that events are produced at a steady rate yet no evidence is given for this. As this is contrary to data given in ONT company presentation which show that dwell time per base is stochastic the authors need to show that this assumption is correct.
9. page 6, "Data Analyses section". The authors say that they do not show reads generated during the first hour are not shown due to various effects. Yet almost a quarter of the reads are generated during this hour. If the authors are saying that reads during this period are substandard and not usable then they should say so. If they are usable then they should analyse them.
10. page 7. results. The authors should say how many flowcells were tested and what % passed the 400 pore minimum threshold.
11. Page 9. "Total event yield". The authors conclude "suggesting data yield is not solely dependent on the number of initial active pores." They should include another possibility, the way ONT measure the number of active pores may not be accurate.
12. Page 14. When talking about base quality the authors should state that this is a Q score.
13. Page 15. "Proportion of 2D pass and fail reads". Users are interested in the overall proportion of passed reads not just the proportion during the first 21 hours. The overall proportion should be stated.
14. Page 17. The authors stated that error estimates are similar for target and control base-calls. They should however point out that E.coli is a neutral GC genome similar to phiX and that other genomes with different base compositions have been reported to give a different error estimate to the lambda control.
15. Page 17. "Correlation between base quality and read accuracy". Because there are so many events on figure 12 A it is impossible to establish if there is really a linear relationship, a gradient of colour intensifying where dots are overlapping would help the reader to see if it's really linear or just all over the place.
16. page 17. The authors state "A theoretical fold coverage of at least 60 was required to call 99.99% of the reference sites accurately from the majority consensus." This is 4 flowcells worth of sequence yet the authors claim a single flowcell to be sufficient in the introduction.
17. page 17. In discussing "Sequence motifs with lower accuracy" the authors should also highlight the fact that homopolymers >5 cannot be resolved and are reported as 5 mers.
18. page 19. In the discussion the authors say "We demonstrated that there was considerable variability in the quality of flow cells" Some figures would be useful here.
19. page 19. In the discussion the authors say "About 32% of the reads from an experiment result in 2D reads from the target genome." They should also state the percentage of 2D pass reads
20. page 19. In the discussion the authors say "When restricted to 2D ‘pass’ reads, the yield decreased to ~12,000 reads containing 75 million bases with a read length distributed centred around 6,700 bases and a mean base quality of 11.9." To put this in context with the previous sentence the authors should state the level of genome coverage that this achieved.
21. page 20. Regarding fast mode. This is already available and giving superior quality data. Thus illustrating whether or not such a consortium can keep up other than perhaps on a blog?
22. page 20. The authors say "Although GC biases may be hard to detect through the sequencing of an E. coli strain with a mean GC content close to 50%, we did not observe a genome-wide GC bias in the 2D reads produced by this platform." In this context they should quote Goodwin *et al.* **who report results from a non-base biased genome, Lver *et al.*
23. Page 20. The authors say "which suggests the MinION itself has some influence on the decrease in base quality over time". Do they mean the minON or the flowcell.
24. Page 21. The authors compare ONT error rates with short read technologies. They should also compare error rate and error profile with PacBio as this is also a long read technology and users would be more interested in that comparison.

We have read this submission. We believe that we have an appropriate level of expertise to confirm that it is of an acceptable scientific standard.
